# Comparative phosphorylation map of Dishevelled 3 links phospho-signatures to biological outputs

**DOI:** 10.1186/s12964-019-0470-z

**Published:** 2019-12-23

**Authors:** Kateřina Hanáková, Ondřej Bernatík, Marek Kravec, Miroslav Micka, Jitender Kumar, Jakub Harnoš, Petra Ovesná, Petra Paclíková, Matěj Rádsetoulal, David Potěšil, Konstantinos Tripsianes, Lukáš Čajánek, Zbyněk Zdráhal, Vítězslav Bryja

**Affiliations:** 1 CEITEC—Central European Institute of Technology, Masaryk University, Kamenice 5, 625 00 Brno, Czech Republic; 2 National Centre for Biomolecular Research, Faculty of Science, Masaryk University, Brno, Czech Republic; 3 Department of Experimental Biology, Faculty of Science, Masaryk University, Kotlářská 2, 611 37 Brno, Czech Republic; 4 Department of Histology and Embryology, Faculty of Medicine, Masaryk University, Brno, Czech Republic; 5 Institute of Biostatistics and Analyses, Faculty of Medicine, Masaryk University, Brno, Czech Republic; 6 Department of Cytokinetics, Institute of Biophysics, Academy of Sciences of the Czech Republic, Brno, Czech Republic

**Keywords:** Dishevelled, DVL3, Phosphorylation, Kinase, Mass spectrometry, CK1, TTBK2, NEK2, Wnt

## Abstract

**Background:**

Dishevelled (DVL) is an essential component of the Wnt signaling cascades. Function of DVL is controlled by phosphorylation but the molecular details are missing. DVL3 contains 131 serines and threonines whose phosphorylation generates complex barcodes underlying diverse DVL3 functions. In order to dissect the role of DVL phosphorylation we analyzed the phosphorylation of human DVL3 induced by previously reported (CK1ε, NEK2, PLK1, CK2α, RIPK4, PKCδ) and newly identified (TTBK2, Aurora A) DVL kinases.

**Methods:**

Shotgun proteomics including TiO_2_ enrichment of phosphorylated peptides followed by liquid chromatography tandem mass spectrometry on immunoprecipitates from HEK293T cells was used to identify and quantify phosphorylation of DVL3 protein induced by 8 kinases. Functional characterization was performed by in-cell analysis of phospho-mimicking/non-phosphorylatable DVL3 mutants and supported by FRET assays and NMR spectroscopy.

**Results:**

We used quantitative mass spectrometry and calculated site occupancies and quantified phosphorylation of > 80 residues. Functional validation demonstrated the importance of CK1ε-induced phosphorylation of S268 and S311 for Wnt-3a-induced β-catenin activation. S630–643 cluster phosphorylation by CK1, NEK2 or TTBK2 is essential for even subcellular distribution of DVL3 when induced by CK1 and TTBK2 but not by NEK2. Further investigation showed that NEK2 utilizes a different mechanism to promote even localization of DVL3. NEK2 triggered phosphorylation of PDZ domain at S263 and S280 prevents binding of DVL C-terminus to PDZ and promotes an open conformation of DVL3 that is more prone to even subcellular localization.

**Conclusions:**

We identify unique phosphorylation barcodes associated with DVL function. Our data provide an example of functional synergy between phosphorylation in structured domains and unstructured IDRs that together dictate the biological outcome.

**Video Abtract.**

**Graphical abstract:**

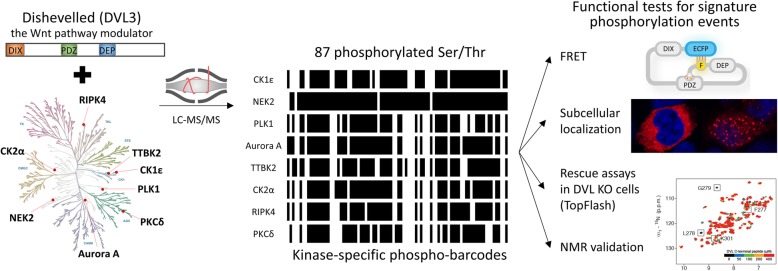

## Background

Wnt signaling pathway has been linked to an etiology of multiple developmental defects, inherited diseases and many types of cancer [1]. Wnt pathways can be divided into several main “branches”. The best studied (canonical) pathway depends on β-catenin and members of the TCF/LEF (T-cell factor/lymphoid enhancer-binding factor) family of transcriptional factors. In the absence of Wnt ligand, the intracellular level of free β-catenin is constantly low due to the activity of a degradation complex including adenomatous polyposis coli, Axin and glycogen synthase kinase (GSK) 3β. Upon Wnt binding, the destruction complex is inhibited allowing accumulation and nuclear activity of β-catenin. However, Wnts can also activate other, so-called non-canonical Wnt pathways, which are β-catenin-independent and biochemically distinct from canonical Wnt signaling. There is strong evidence that several such pathways exist (for complete overview see [2]).

All known Wnt-induced pathways are transduced through Frizzled transmembrane receptors [3] and intracellular protein Dishevelled (Dsh in *Drosophila*, DVL1–3 in human). DVL proteins consist of three structured domains: N-terminally located DIX domain, centrally located PDZ domain and C-terminally located DEP domain. The individual domains are linked by intrinsically disordered regions followed by approximately 200 amino acid long, also intrinsically disordered, C-terminal tail.

There is a general agreement based on genetic experiments that DVL plays a crucial role as a signaling hub in both Wnt/β-catenin and non-canonical Wnt pathways [4]. In addition DVL has been reported to have multiple other functions such as docking of basal body [5, 6], function and maintenance of primary cilia [7], cytokinesis [8], or positioning of the mitotic spindle [9]; all of them possibly linked to its role in the regulation of centrosomal cycle [10].

Despite the well documented role of DVL in the Wnt signaling and the growing evidence for its participation in additional cellular processes, the molecular mechanisms that regulate DVL action in the cell are almost unknown. However, it is believed that post-translational modifications (PTM), particularly phosphorylation, represent a key component of such regulatory mechanisms. DVL proteins are very rich in serine (S) and threonine (T); for example, DVL3 contains 131 S/T residues, which can be potentially phosphorylated. The best described consequence of the activation of both Wnt/β-catenin as well as non-canonical Wnt pathway is phosphorylation of DVL by the Wnt-induced Casein kinase 1 ɛ (CK1ɛ) [11–14]. In addition to CK1ε, multiple other kinases have been reported to phosphorylate DVL in different contexts. For example, CK2α in both Wnt signaling pathways [14–16], Polo-like kinase (PLK) 1 in the control of mitotic spindle [9], Nima-related kinase (NEK) 2 in the centrosome [10, 17], protein kinase C (PKC) δ in non-canonical Wnt signaling [18], and receptor-interacting protein kinase (RIPK) 4 in the Wnt/β-catenin signaling [19]. Recent work using mass spectrometry [10, 20, 21] has identified more than 50 S/T of DVL that are indeed phosphorylated. However, the functional significance of these phosphorylation sites in DVL remains unclear. With respect to the current understanding of PTMs in the intrinsically disordered proteins, it is reasonable to speculate that the physiological function is achieved by a combination (“barcode”) of phosphorylated sites rather than by mere phosphorylation of individual sites.

In this study we analyzed and compared the phosphorylation of DVL3 induced by eight S/T kinases. We have selected DVL3 as a candidate because its absence shows the strong phenotypes in vivo [22]. We have studied the phosphorylation of this DVL isoform earlier [10, 16, 23, 24], which allows for a direct comparison. The studied kinases included six previously reported DVL kinases, Aurora A that was reported with DVL in the same complex [25] and TTBK2, a DVL kinase identified in this study. We have applied complementary proteomic approaches to describe in detail and in a quantitative manner how individual kinases modify DVL3. The phosphorylation barcoding unveiled unique but also common phosphorylation patterns that represent a reference point for the interpretation of existing and any future data analyzing phosphorylation of DVL. Last but not least, our work provides an example of universal pipelines for the phosphoanalysis of proteins modified in complex patterns at dozens of residues.

## Materials and methods

### Cell culture, transfection, and treatments

HEK293T, HEK293 T-REx and DVL1/2/3-null HEK293 T-REx cells were propagated in DMEM, 10% FCS, 2 mM L-glutamine, 50 units/ml penicillin, 50 units/ml streptomycin. CK1ε inhibitor PF-670462 was used at 10 μM. Cells were seeded on appropriate culture dishes (15 cm diameter dish for IP, 24-well plate for WB, dual luciferase assay, ICC) and the next day were transfected using polyethylenimine (PEI) in a stoichiometry of 3 μl PEI (0.1% w/v in MQ water) per 1 μg of DNA. Cells were harvested for immunoblotting or immunocytofluorescence 24 h after transfection, for immunoprecipitation after 48 h. The following plasmids have been published previously: FLAG-DVL3 [26], CK1ε [27], Myc-NEK2 [28], FLAG-PLK1 [29], Myc-Aurora A [30], HA-CK2α [31], YFP-PKCδ [32], VSV-RIPK4 [33] and GFP-TTBK2 WT and KD [34].

### Dual luciferase TopFlash/Renilla reporter assay

**Fig. 1:**
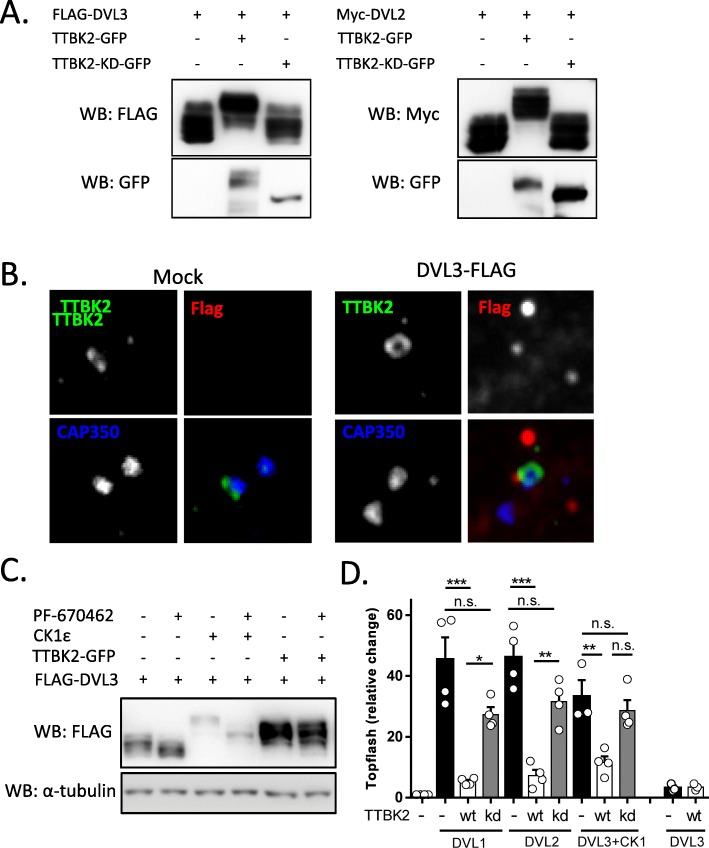
Identification of TTBK2 as a novel DVL kinase. **a**: HEK293 cells were transfected with FLAG-DVL3 and Myc-DVL2 plasmids with wild type (wt) or kinase dead (KD) TTBK2-GFP. Active TTBK2 promoted phosphorylation-dependent mobility shift of DVL3 on Western blotting. **b**: Endogenous TTBK2 (green) localized into distal appendages of the mother centriole in hTERT-RPE1 cells (left). Overexpression of FLAG-DVL3 (stained in red) was not able to displace TTBK2 from the centriole (right). Centrioles were stained with CAP350 (blue). **c**: HEK293 cells were transfected with indicated plasmids, treated with CK1ε inhibitor PF-670462 (10 μM) and subsequently analyzed by Western blotting. TTBK2-induced electrophoretic mobility shift of DVL3 was not diminished upon CK1ε inhibition unlike the mobility shift induced by CK1ε. **d**: HEK293 cells were transfected with indicated plasmids and by the TopFLASH reporter system. Luminescence in the cell lysates was measured 24 h after transfection. Mean, SD and individual data points are indicated. Statistical differences were tested by One-way ANOVA and Tukey’s post test (* *p* < 0.05, ** *p* < 0.01, *** *p* < 0.001)

For the luciferase reporter assay, cells were transfected with 0.1 μg of Super8X TopFlash construct, 0.1 μg of pRLTKluc (Renilla) luciferase construct and other plasmids as indicated (50 ng of DVL3 in Fig. 1d, 200 ng of DVL3 in Fig. 9b) in a 24-well plate and processed 24 h after transfection. For the TopFlash assay, a Promega dual luciferase assay kit was used according to the manufacturer’s instructions. Relative luciferase units of firefly luciferase were measured and normalized to the Renilla luciferase signal.

### Coimmunoprecipitation and Western blotting

For MS/MS-based identification of phosphorylation, HEK293T cells were seeded on 15 cm dishes and transfected with corresponding plasmids (8 μg of DVL3 plasmid plus 8 μg of pCDNA3 or plasmid encoding the kinase per dish) 24 h after seeding. Two ml of ice-cold lysis buffer supplemented with protease inhibitors (Roche Applied Science, 11,836,145,001), phosphatase inhibitors (Calbiochem, 524,625), 0.1 mM DTT, and 10 mM N-ethylmaleimide (Sigma E3876) was used for lysis of one 15 cm dish. Lysate was collected after 20 min of lysis on 4 °C and was cleared by centrifugation at 18000×g for 20 min. Three μg of anti-FLAG M2 (F1804; Sigma) antibody were used per sample. Samples were incubated with the antibody for 40 min, then 25 μl of G protein-Sepharose beads (GE Healthcare, 17–0618-05) equilibrated in the lysis buffer were added to each sample. Samples were incubated on the carousel overnight, washed 6 times with lysis buffer and finally, 40 μl of 2× Laemmli buffer was added and samples were subsequently boiled.

The samples were loaded to 8% SDS-PAGE gels. Electrophoresis was preformed through the stacking gel at 100 V and through the separating gel at 150 with PageRuler Prestained Protein Ladder (Thermofisher, Cat. No. 26620) as a marker. Gels were then process for mass spectrometry or the proteins were transferred to the polyvinylidene difluoride membrane (Merck Millipore, Cat. No. IPVH00010) by Western Blotting (WB). Subsequently, the membrane was blocked in 5% defatted milk or 3% BSA for 1 h with shaking. After the blocking step, the membrane was incubated with corresponding primary and secondary antibodies. Proteins were visualized by ECL (Enhanced chemiluminescence; Merck Millipore, Cat. No. WBKLS0500). The signal was detected using Vilber FUSION-SL system. Western blot was quantified using ImageJ software.

The antibodies used were: anti-FLAG M2 (Sigma-Aldrich, #F1804) for WB and IP, anti-CK1ɛ (Santa Cruz, #sc-6471), anti-GFP (Fitzgerald, #20R-GR-011), HA11 (Covance #MMS-101R), VSV (Sigma-Aldrich #V 5507), c-Myc (Santa Cruz, #sc-40), α-tubulin (Proteintech, 66,031–1-Ig), anti-DVL3 (sc-8027, Santa Cruz Biotechnology) and anti-β-actin (cs4970, Cell Signalling). Following phospho-specific antibodies have been published previously – pS280-DVL3 (pS280) [10], pS643 [20], and pS697 [10]. Anti phospho-S192 antibody was prepared by immunizing rabbits by TTSFFDS(p) DEDDST peptide on a service basis by Moravian Biotechnology (http://www.moravian-biotech.com).

### Immunofluorescence

Cells were seeded onto glass coverslips and next day transfected according to the scheme. 24 h post transfection medium was removed, cells were washed by PBS and fixed by 4% PFA for 10 min or ice-cold MetOH (5 min/− 20 °C, Fig. 1b). Coverslips were then washed in PBS and incubated with primary antibodies for 1 h, washed three times with PBS and then incubated with secondary antibodies conjugated to Alexa Fluor 488 (Invitrogen A11001) or/and Alexa Fluor 594 (Invitrogen A11058), washed with PBS and stained with DAPI (1:5000); all coverslips were mounted on microscopic slides. Cells were then visualized on Olympus IX51 fluorescent microscope using 40× air or 100× oil objectives and/or Olympus Fluoview 500 confocal laser scanning microscope IX71 using 100× oil objective. 200 positive cells per experiment (*N* = 3) were analyzed and scored according to their phenotype into two categories (punctae/even). The antibodies used were as follows: anti-FLAG M2 (Sigma-Aldrich, #F1804), anti-DVL3 (Santa Cruz, #sc-8027), anti-CK1ɛ (Santa Cruz, #sc-6471), HA11 (Covance #MMS-101R), VSV (Sigma-Aldrich #V 5507), c-Myc (Santa Cruz, #sc-40) and anti-GFP (Fitzgerald, #20R-GR-011), CAP350 [35], TTBK2 (Sigma-Aldrich, #HPA018113). Images presented in Fig. 1b were acquired using DeltaVision-Elite system (Applied Precision/GE) with a 100×/1.4 Apo plan oil immersion objective. Image stacks were taken with a z distance of 0.2 μm, deconvolved (conservative ratio, three cycles), and projected as maximal intensity images by using SoftWoRX (Applied Precision/GE).

### FlAsH FRET

The HEK293 cells were seeded onto round 24-mm cover slips, which were previously placed in six-well plates and coated with 200 μl of poly-D-lysine (1 mg/ml) for 20 min. Cells were transfected 16–18 h later using Effectene (Qiagen), according to the manufacturer’s instructions. DMEM was replaced 6 h later and the analysis was done 24 h after transfection.

FlAsH labeling of the DVL3 FlAsH III sensor was performed as previously described [24]. Shortly, transfected cells were washed once with Hank’s Balanced Salt Solution (HBSS) containing 1.8 g/l glucose and then incubated at 37 °C for 1 h with HBSS supplemented with 500 nM FlAsH; 12.5 μM 1,2-ethanedithiol (EDT). In order to reduce non-specific labeling, cells were then rinsed once with HBSS and incubated for 10 min with HBSS containing 250 μM EDT and corresponding inhibitors. Finally, cells were washed twice with HBSS and maintained in DMEM medium.

To determine the FRET efficiency of the DVL3 FlAsH III sensor, coverslips with the cells were mounted using an Attofluor holder (Molecular Probes) and placed on a Zeiss inverted microscope (Axiovert200), equipped with 63x oil objective lens and a dual-emission photometric system (Till Photonics). Cells were excited at 436 ± 10 nm using a frequency of 10 Hz with 40 ms illumination time out of a total of 100 ms. Emission of ECFP (480 ± 20 nm) and FlAsH (535 ± 15 nm), and the FRET ratio (FlAsH/ECFP) were monitored simultaneously. Fluorescence signals were detected by photodiodes and digitalized using an analogue-digital converter (Digidata 1440A, Axon Instruments)^.^ Fluorescence intensities data were acquired using Clampex software (Axon Instruments). During measurements, cells were maintained in imaging buffer; 5 mM of 2,3-dimercapto-1-propanol (also called British anti-Lewisite – BAL) was added to the cells approximately 40 s after the recording started. Recovery of ECFP fluorescence was monitored over time and FRET efficiency was calculated as described [36]. One independent experiment represents approximately 4–6 repeats (i.e. single-cell FRET signals) for each condition.

### Site directed mutagenesis

The mutagenesis reactions were performed using the QuikChange II XL Site-Directed Mutagenesis Kit following the manufacturer’s instructions (Agilent Technologies, #200521). All mutations described in this study were verified by Sanger sequencing. Following primers were used:

**Table:** 

DVL3 S268A forw	ATCTCCATTGTGGACCAAGCCAACGAGCGTGGTGACGGC
DVL3 S268A rev	GCCGTCACCACGCTCGTTGGCTTGGTCCACAATGGAGAT
DVL3 S311A for	ATCAACTTTGAGAACATGGCTAATGACGATGCAGTCCGG
DVL3 S311A rev	CCGGACTGCATCGTCATTAGCCATGTTCTCAAAGTTGAT
DVL3 S268E forw	ATCTCCATTGTGGACCAAGAGAACGAGCGTGGTGACGGC
DVL3 S268E rev	GCCGTCACCACGCTCGTTCTCTTGGTCCACAATGGAGAT
DVL3 S311E forw	ATCAACTTTGAGAACATGGAGAATGACGATGCAGTCCGG
DVL3 S311E rev	CCGGACTGCATCGTCATTCTCCATGTTCTCAAAGTTGAT
Dvl2 S281E forw	TACAACTTCCTGGGTATCGAGATTGTTGGCCAGAGCAAT
Dvl2 S281E rev	ATTGCTCTGGCCAACAATCTCGATACCCAGGAAGTTGTA
Dvl2 S298E forw	GGCGGCATCTACATTGGCGAGATCATGAAGGGTGGGGCC
Dvl2 S298E rev	GGCCCCACCCTTCATGATCTCGCCAATGTAGATGCCGCC

### Rescue assays

For transfection HEK293 T-REx (WT) and DVL1/2/3-null HEK293 T-REx cells (D1/2/3 TKO HEK293 T-REx) cells were seeded at 200000 cell/well in 24 well plate density. Next day, the cells were transfected using polyethylenimine (PEI) in concentration 1 μg/ml and pH 7.4 and the PEI ratio 6 μl of PEI/ 1 μg DNA. Mixture of transfected plasmids (10 ng of indicated DVL3 plasmid, 100 ng of Super8X TopFlash construct and 100 ng of pRLTKluc (Renilla) luciferase construct per well, the total amount of DNA was equalized by pcDNA3.1 to 400 ng DNA/well) and PEI was diluted separately in plain DMEM (DMEM without FBS, L-glutamine and antibiotics). After 6 h, medium containing transfection mix was removed and exchanged by complete DMEM medium. In all experiments cells were treated by 1 μM porcupine inhibitor LGK974 (Stem RD, 974–02) to reduce the autocrine secretion of all Wnt ligands and recombinant human R-spondin1 250 ng/ml (PeproTech, 120–38). For stimulation of the cells, Wnt3a recombinant protein was used for 14 h in concentration 80 ng/ml (R&D Systems, 5036-WN-CF). Control stimulations were done with 0.1% BSA in PBS. Samples were then analyzed by TopFlash reporter assay and WB.

### Protein expression and purification

Human DVL2 PDZ domain (aa 265–361) and its phosphomimicking mutant (S281E + S298E) were cloned into pET vector with N-terminal His_6_-tag_,_ a lipoyl domain tag and a TEV cleavage site. All proteins were expressed in BL21-DE3(RIL) cells. For NMR studies, cells were grown in minimal medium (M9) supplemented by ^15^NH_4_Cl (1 g/l) and/or ^13^C_6_ glucose (2 g/l) and induced with 0.5 mM IPTG for 24 h at 16 °C. Proteins were purified as described before [24] and stored in 20 mM Hepes (pH 6.8) and 50 mM KCl for NMR studies. The DVL3_C peptide 698–716 with phosphorylated S700 (DVL_C) used in NMR studies was purchased from KareBay Biochem, Inc. (New Jersey, USA).

### NMR spectroscopy

NMR experiments were carried out at CEITEC Josef Dadok National NMR Centre on a 700 MHz Bruker Avance III spectrometers equipped with ^1^H/^13^C/^15^N TCI cryogenic probe head with *z*-axis gradients. Chemical shift assignments of the phosphomimicking PDZ mutant (S281E + S298E) were obtained automatically using 4D-CHAINS technology [37] as described before for the wt PDZ domain [24]. NMR titrations were performed in series of ^1^H-^15^N HSQC spectra using 100 μM of ^15^N-labeled protein (wild type or phosphomimicking mutant) and increasing amounts of DVL_C (stock concentration of 800 μM).

### Mass spectrometry

#### In gel digestion

Immunoprecipitates were separated on SDS-PAGE gel electrophoresis, fixed with acetic acid in methanol and stained with Coomassie brilliant blue for 1 h. Corresponding 1D bands were excised. After destaining, the proteins in gel pieces were incubated with 10 mM DTT at 56 °C for 45 min. After removal of DTT excess samples were incubated with 55 mM IAA at room temperature in darkness for 30 min, then alkylation solution was removed and gel pieces were hydrated for 45 min at 4 °C in digestion solution (5 ng/μl trypsin, sequencing grade, Promega, in 25 mM AB). The trypsin digestion proceeded for 2 h at 37 °C on Thermomixer (750 rpm; Eppendorf). Subsequently, the tryptic digests were cleaved by chymotrypsin (5 ng/μl, sequencing grade, Roche, in 25 mM AB) for 2 h at 37 °C. Digested peptides were extracted from gels using 50% ACN solution with 2.5% formic acid (FA) and concentrated in speedVac concentrator (Eppendorf). The aliquot (1/10) of concentrated sample was transferred to LC-MS vial with already added polyethylene glycol (PEG; final concentration 0.001%, [38] and directly analyzed by LC-MS/MS for protein identification.

#### Phosphopeptide enrichment

The rest of the sample (9/10) was used for phosphopeptide analysis. Sample was diluted with acidified acetonitrile solution (80% ACN, 2% FA). Phosphopeptides were enriched using Pierce Magnetic Titanium Dioxide Phosphopeptide Enrichment Kit (Thermo Scientific, Waltham, Massachusetts, USA) according to manufacturer protocol and eluted into LC-MS vial with already added PEG (final concentration 0.001%). Eluates were concentrated under vacuum and then dissolved in water and 0.6 μl of 5% FA to get 12 μl of peptide solution before LC-MS/MS analysis.

#### LC-MS/MS analysis

LC-MS/MS analyses of peptide mixture were done using RSLCnano system connected to Orbitrap Elite hybrid spectrometer (Thermo Fisher Scientific) with ABIRD (Active Background Ion Reduction Device; ESI Source Solutions) and Digital PicoView 550 (New Objective) ion source (tip rinsing by 50% acetonitrile with 0.1% formic acid) installed. Prior to LC separation, peptide samples were online concentrated and desalted using trapping column (100 μm × 30 mm) filled with 3.5 μm X-Bridge BEH 130 C18 sorbent (Waters). After washing of trapping column with 0.1% FA, the peptides were eluted (flow 300 nl/min) from the trapping column onto Acclaim Pepmap100 C18 column (3 μm particles, 75 μm × 500 mm; Thermo Fisher Scientific) by 65 min long gradient. Mobile phase A (0.1% FA in water) and mobile phase B (0.1% FA in 80% acetonitrile) were used in both cases. The gradient elution started at 1% of mobile phase B and increased from 1 to 56% during the first 50 min (30% in the 35th and 56% in 50th min), then increased linearly to 80% of mobile phase B in the next 5 min and remained at this state for the next 10 min. Equilibration of the trapping column and the column was done prior to sample injection to sample loop. The analytical column outlet was directly connected to the Digital PicoView 550 ion source.

MS data were acquired in a data-dependent strategy selecting up to top 10 precursors based on precursor abundance in the survey scan (350–2000 m/z). The resolution of the survey scan was 60,000 (400 m/z) with a target value of 1 × 10^6^ ions, one microscan and maximum injection time of 200 ms. High resolution (15,000 at 400 m/z) HCD MS/MS spectra were acquired with a target value of 50,000. Normalized collision energy was 32% for HCD spectra. The maximum injection time for MS/MS was 500 ms. Dynamic exclusion was enabled for 45 s after one MS/MS spectra acquisition and early expiration was disabled. The isolation window for MS/MS fragmentation was set to 2 m/z.

#### Data analysis

The analysis of the mass spectrometric RAW data was carried out using the Proteome Discoverer software (Thermo Fisher Scientific; version 1.4) with in-house Mascot (Matrixscience; version 2.4.1) search engine utilization. MS/MS ion searches were done against in-house database containing expected protein of interest with additional sequences from cRAP database (downloaded from http://www.thegpm.org/crap/). Mass tolerance for peptides and MS/MS fragments were 7 ppm and 0.03 Da, respectively. Oxidation of methionine, deamidation (N, Q) and phosphorylation (S, T, Y) as optional modification, carbamidomethylation of C as fixed modification, TrypChymo enzyme specifity and three enzyme miss cleavages were set for all searches. The phosphoRS (version 3.1) feature was used for preliminary phosphorylation localization. Final localization of all phosphorylations (including those with ambiguous localization) was performed by manual evaluation of the fragmentation spectra of the individual phosphopeptides. Based on the presence of individual fragments in the peptide sequence, it was decided whether the localization was accurate or not.

Quantitative information was assessed and manually validated in Skyline software (Skyline daily 3.6.1.10230). Normalization of the data was performed using the set of phosphopeptide standards (added to the sample prior phosphoenrichment step; MS PhosphoMix 1, 2, 3 Light, Sigma-Aldrich) and by non-phosphorylated peptides identified in direct analyses.

All quantitative data (peptide intensities) were processed by two approaches (see Fig. 3a). In the first approach (pipeline #1 and #3) that often resulted in the formation of phosphorylated clusters all identified phosphorylated peptides were considered. This has resulted in three categories of identification: (i) one peptide or set of peptides with one clearly localized phosphorylated site, (ii) one or set of overlapping peptides covering sequence region with two or more clearly localized phosphorylated sites (phosphorylated sites separated by a comma in the Figures) and (iii) one or set of overlapping peptides covering sequence region with two or more phosphorylated sites, but some of them are not clearly localized (phosphorylated sites separated by a dash in the Figures). Sum of intensities of phosphorylated peptides (includes different peptide sequences, charges and peptides with other modifications) was calculated for each cluster. In case of direct analysis (pipeline #1), we calculated site occupancies as percentage ratio of phosphorylated peptide intensity (summed if more than one) to total intensity (summed intensities of phosphorylated peptide(s) + corresponding non-phosphorylated peptide(s)). Sites/clusters with the site occupancy > 5% at least in one experiment are shown in Fig. 4. For pipeline #3, the difference between the phosphorylated peptide summed intensity in the kinase-induced sample and the control (in log10 scale) was calculated for each replicate and the mean from all three replicates was used for the heat map (see in Additional file 2: Figure S2). In the second approach (pipeline #2), only phosphorylated peptides with the clearly localized phosphorylation site (based on manual inspection) were considered. In case of the multiphosphorylated peptides the total intensity of the peptide was assigned to each phosphorylated site. Sum of intensities of phosphorylated peptides was calculated for each phosphorylated site. The heat map (Fig. 5) was build-up in the same way as for pipeline #3 described above. For the production of phosphoplots the sum of intensities for each phosphorylated site (pipeline #2) were log-transformed (log10) and average from 3 replicates was calculated (values under the detection limit were not included in the calculation).

### Numerical data & statistics

#### Cluster analysis

Data of absolute peptide intensities were log-transformed (log10) because of their log-normal distribution. Log-transformed data of each replicate were standardized to control (kinase subtracted from “no kinase” control) and averaged from 3 replicates. Cluster analysis was applied both for kinases and for phosphosites; Ward’s minimum variance method based on the Lance-Williams recurrence and Euclidean distance were used. Circular plot accompanied by heat map was used for visualization of complex relation among phosphosites and kinases. All analyses were performed using R software.

#### Other analyses

One-way ANOVA and Tukey Post tests were calculated by GraphPad Prism (GraphPad Software Inc.).

## Results

### Identification of TTBK2 as a kinase acting upstream of DVL

Our previous work identified interactions between DVL3, CEP164, and TTBK2 kinase [34, 39]. To examine the possible regulation of DVL by TTBK2, we tested whether this poorly characterized centrosomal kinase phosphorylates DVL3 and DVL2 in HEK293 cells. As shown in Fig. 1a, TTBK2 co-expression induced a prominent electrophoretic mobility shift for both DVL3 and DVL2 in a kinase activity-dependent manner, suggesting that TTBK2 is capable to efficiently promote phosphorylation of DVL. TTBK2 also gets auto-phosphorylated as described earlier [34]. TTBK2 has been described as a kinase that primarily resides on the mother centriole where it regulates ciliogenesis [34, 40–45]. Given the previous reports on DVL centrosomal localization and the possible DVL implication in ciliogenesis [6, 7, 10, 46] we tested whether overexpressed DVL has the capacity to displace TTBK2 from the centriole. As shown in Fig. 1b (left panel), we confirmed, in line with earlier reports, the localization of TTBK2 to the mother centriole, which is however not affected by overexpression of DVL3 (Fig. 1b, right).

Mobility-shift of DVL2 and DVL3 induced by TTBK2 (Fig. 1a) can be in principle a consequence of direct phosphorylation of DVL by TTBK2 or a consequence of activation of other DVL kinases by TTBK2. In the second scenario, the well-established DVL kinase CK1ε represents the most obvious candidate target of TTBK2. To address whether TTBK2 acts directly on DVL or rather acts as CK1ε activator we treated cells with the CK1 inhibitor PF-670462 and subsequently analyzed the electrophoretic mobility shift of DVL3. As shown in Fig. 1c, PF-670462 efficiently reduced phosphorylation induced by CK1ε but not by TTBK2, hence demonstrating that TTBK2 phosphorylation of DVL does not require CK1ε activity.

The best-defined role of DVL is the positive regulation of the Wnt/β-catenin pathway. In order to address if TTBK2 modulates this DVL function we analyzed the ability of TTBK2 to promote or to inhibit DVL-induced TCF/LEF-dependent luciferase reporter (TopFlash) in HEK293 cells. Interestingly, TTBK2 did inhibit efficiently DVL1- and DVL2-induced TopFlash activation in a kinase activity-dependent manner. Because DVL3 alone induces TopFlash very poorly (Fig. 1d, right; see also [47]) we co-transfected CK1ε and DVL3 and even in this case TTBK2 co-expression reduced TopFlash activation (Fig. 1d). In summary, we demonstrate that the robust phosphorylation of DVL mediated by TTBK2 is associated with the decreased capacity of DVL to act in the Wnt/β-catenin pathway.

### Design and validation of DVL kinase panel

**Fig. 2:**
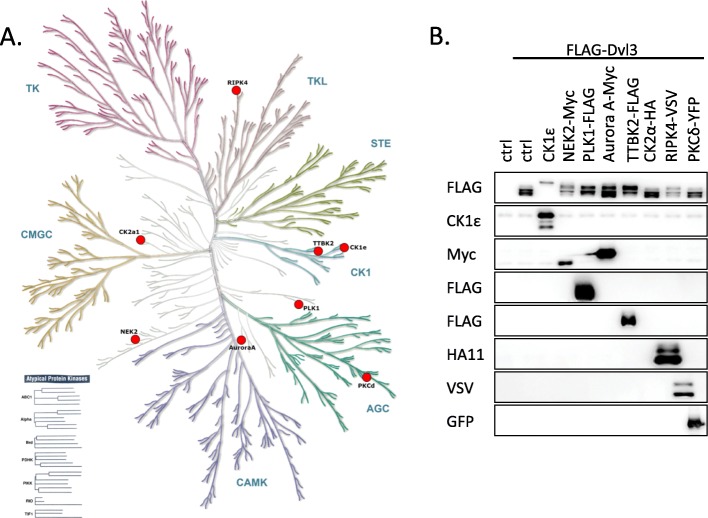
Validation of the panel of DVL3 kinases. **a**: Visualization of the kinases used in this study in the phylogenetic kinome tree (http://www.kinhub.org/kinmap/). The individual kinases are representatives of distant kinase groups except for CK1ε and TTBK2 that are members of CK1 superfamily. **b**. HEK293 cells were transfected by plasmids encoding FLAG-DVL3 and the indicated kinase. Ability of individual kinases to promote DVL3 phosphorylation detected as the electrophoretic mobility shift on WB was assayed. Alpha-tubulin was used as a loading control

TTBK2 represents an additional kinase to the ever-growing list of DVL kinases. Currently, multiple kinases from diverse families have been reported to phosphorylate at least one of DVL isoforms. From the fragmented published results, it is not possible to find out the unique/general or constitutive/induced phosphorylation events and patterns that are associated with various functions of DVL in connection to individual kinases. This prompted us to perform a direct comparison of DVL phosphorylation by individual kinases. We chose human HEK293 cells, a common model for the analysis of Wnt signaling, and human DVL3 as a representative DVL protein. Previously reported DVL kinases – CK1ε [11], CK2α [15], PLK1 [9], NEK2 [10, 17], PKCδ [18], RIPK4 [19] and the newly identified TTBK2 were added to the panel. We also included mitotic kinase Aurora A that has been reported to act in the same complex with DVL [25]. These S/T kinases represent very diverse members of the protein kinase family as visualized on the kinome tree (Fig. 2a). Most kinases – except for CK2α and PKCδ – could trigger electrophoretic mobility shift of DVL3 when co-expressed with DVL3 (Fig. 2b).

### Pipelines for the generation of the phosphorylation map of DVL3

**Fig. 3:**
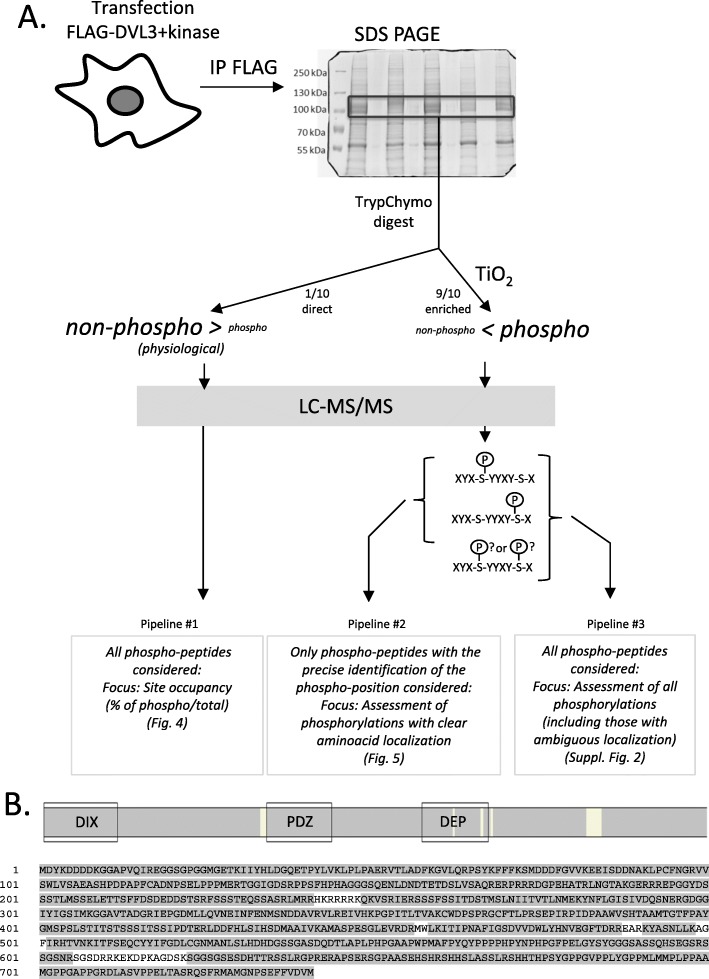
Experimental design. **a**: FLAG-DVL3 was overexpressed (with or without kinase) in HEK293 cells. After cell lysis DVL3 was immunoprecipitated using anti-FLAG antibody. Immunoprecipitates were separated on SDS-PAGE gel electrophoresis, stained with Coomassie brilliant blue and the 1D bands corresponding to DVL3 were excised, digested with trypsin and subsequently cleaved by chymotrypsin. In the pipeline #1, the aliquot (1/10) of concentrated sample was directly analyzed by LC-MS/MS in order to analyze site occupancy of the abundant phosphorylated sites. The rest of the sample was enriched for phosphorylated peptides using TiO_2_ and analyzed by LC-MS/MS to obtain detailed information of DVL3 phosphorylation status. Data from LC-MS/MS were searched, manually validated in Skyline software and further processed by two approaches. In the first approach (pipeline #2) only phosphorylated peptides with the clearly localized phosphorylated site (based on manual inspection of spectra) were considered. In the second approach (pipeline #3) we have considered all phosphorylated peptides that in some cases resulted in the formation of “clusters” of phosphorylated sites. **b**: The overall sequence coverage of DVL3 across all kinases and replicates. Regions of DVL3 covered by the peptides detected (Mascot score > 20) in any of the MS/MS analyses are highlighted in grey. For sequence coverage in individual samples see Additional file 6: Table S2

Following validation of our experimental system (Fig. 2) we performed a global analysis of DVL3 phosphorylation events. In three independent experiments, FLAG-DVL3 was overexpressed in HEK293 cells, with or without the studied kinase, immunoprecipitated using anti-FLAG antibody, separated on SDS-PAGE and stained with Coomassie Brilliant Blue (see Additional file 1: Figure S1 for the gels used in this study). Bands corresponding to DVL3 were cut out and after TrypChymo digestion 1/10 of the sample was analyzed directly (see pipeline #1 in Fig. 3a) and the remaining 9/10 of the peptide mixture was enriched for the phosphorylated peptides by TiO_2_ (see pipelines #2 and #3). All samples were subsequently analyzed by liquid chromatography coupled to mass spectrometry (LC-MS/MS).

Peptides with accurately characterized phosphorylated site(s) were identified alongside with peptides of ambiguous phospholocalization, as it is common to proteomic studies. Therefore, in the accurate position focused analysis (pipeline #2) only a subgroup of peptides with clearly identified phosphorylated positions (based on manual inspection) were included. In the overall quantitative analysis (pipeline #3) all phosphorylated peptides were processed despite the uncertainty of phosphoresidues and as such were presented as “phosphorylated clusters”. For the detailed information on the quantification in these two pipelines see Materials and Methods.

Combination of the above-mentioned approaches allowed us to cover more than 95% of the DVL3 sequence across all experiments (Fig. 3b); for sequence coverage in individual samples see Additional file 6: Table S2.

### Phosphorylation map of DVL3: site occupancy of phosphorylated sites

To assess the site occupancy, we utilized direct analysis of the samples without any enrichment (the experimental pipeline #1, Fig. 3a) which allowed us to detect phosphorylated and non-phosphorylated peptides corresponding to the same position(s). Subsequently we calculated the approximate occupancy of the selected phosphorylated sites, i.e. % of DVL3 molecules phosphorylated at given S or T residue(s).

**Fig. 4:**
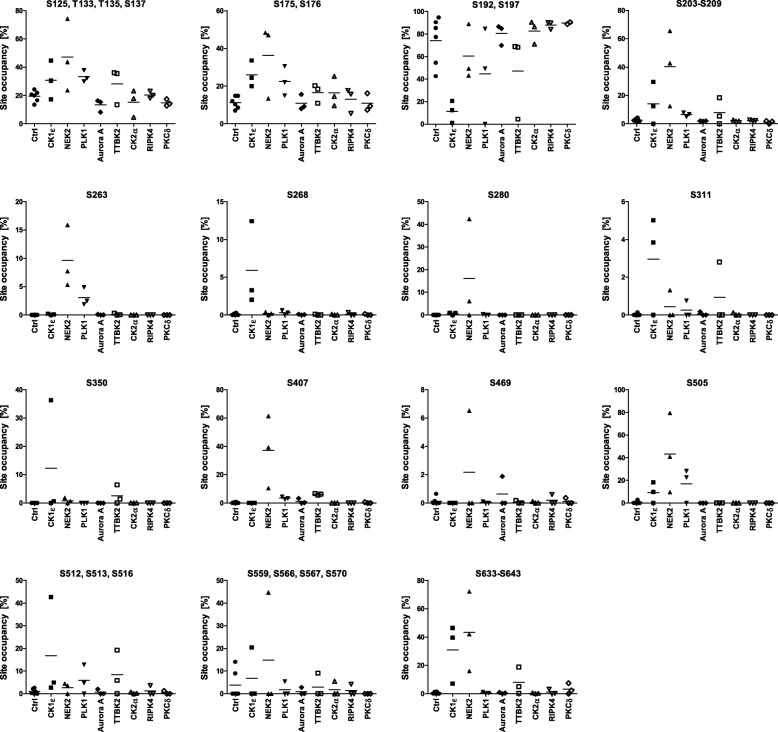
Site occupancy of the abundant phosphorylated sites. Site occupancy analysis was performed according to the pipeline #1 in Fig. 3a. Fifteen phosphorylated peptides or clusters that were phosphorylated in more than 5% at least in one replicate are plotted. Graphs present individual data points from three biological replicates (two controls/biological sample) and the mean values (horizontal line)

We calculated site occupancies as the percentage ratio of phosphorylated peptide intensity (or sum of intensities if more than one) covering individual phosphosites or clusters to total intensity (phosphorylated peptide(s) + corresponding non-phosphorylated peptide(s)). Phosphorylated sites/clusters with > 5% site occupancy in one sample are plotted in Fig. 4. For three clusters (S232–S244, T608–S612, S622–S630) we were not able to detect matching non-phosphorylated peptides and therefore these sites were not included in the site occupancy calculations.

We observed an increase in the phosphorylated site occupancy (above 5%) after overexpression of at least one protein kinase in all but one [14] cases (Fig. 4). The notable exception was the phosphorylated cluster S192, S197, whose phosphorylation decreased in the presence of CK1ε and to a lesser extent in the presence of TTBK2, PLK1 and NEK2. Phosphorylation of three sites was induced selectively only by one kinase; S280 and S407 were induced only by NEK2, and S268 was induced only by CK1ε. In the remaining 11 sites/clusters, two or three kinases were capable to trigger phosphorylation: CK1ε/TTBK2 at S350 and at the cluster S512, S513, S516; NEK2/PLK1 at S263; NEK2/Aurora A at S469 (in the single replicate); NEK2/PLK1/CK1ε at S505; NEK2/CK1ε/TTBK2 (and to some extent PLK1) at S311, and at the clusters S203–S209 and S633–S643. Three other clusters – cluster S125, T133, S135, S137, cluster S175, S176 and cluster S559, S566, S567, S570 showed relatively high occupancy in the control but were further phosphorylated by several kinases, including NEK2, CK1ε, TTBK2 and PLK1.

### Phosphorylation map of DVL3: detailed analysis after phospho-enrichment

**Fig. 5:**
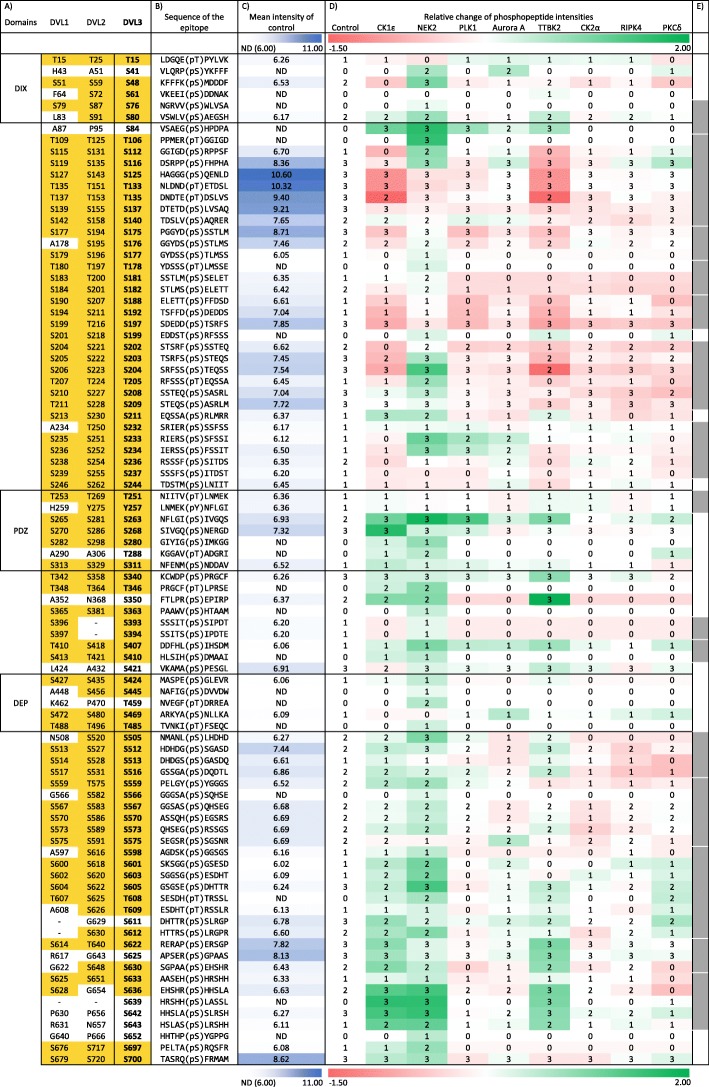
Phosphorylation map of DVL3. All identified phosphorylation sites obtained from the pipeline #2 are visualized as a heatmap. Color intensities reflect relative change in the site phosphorylation (red – decrease, green – increase). Following additional information is also provided: **a**) Corresponding sites in human DVL1 and DVL2. Positions conserved in DVL1 and/or DVL2 either as Ser or Thr are highlighted in yellow. Position of the structured domains (DIX, PDZ and DEP) is indicated. **b**) The sequence of the phosphorylated epitope. Five amino acids before and after the identified phosphorylated site are shown. **c**) Mean absolute intensities of the phosphorylation sites in the control (DVL3 without exogenous kinase; *N* = 6) are expressed in the shades of blue. Numbers indicate decadic logarithm of the mean. ND indicated in white corresponds means “not detected”. All signals lower than 1 × 10^6^, corresponding to log value 6.0 were considered as not detected. **d**) Nine columns represent heat map of relative change of phosphorylated peptide intensities (in log10 scale) obtained for individual kinases (relative to control). Numbers in the heatmap fields (0, 1, 2, 3) indicate the number of experimental replicates with the positive identification of the given phosphorylated site. **e**) Grey boxes indicate the position of clusters of sites analyzed also according to the pipeline #3 in the Additional file 2: Figure S2

In order to analyze phosphorylation of DVL3 in depth, we enriched the phosphorylated peptides by TiO_2_. This approach, commonly utilized for detailed screening of protein phosphorylation, allows detection of less abundant phosphopeptides. Phospho-enrichment data were processed in two ways (Fig. 3a). Primarily, we assessed qualitatively and quantitatively only phosphopeptides with clearly identified (validated by manual inspection of MS/MS data) phosphorylation site(s) (the experimental pipeline #2, Fig. 3a). By this approach we detected 88 unique phosphorylation sites in DVL3. Peptide intensities (Additional file 5: Table S1) were compared with the DVL3-only control dataset in order to express the relative increase/decrease in the phosphorylation of each site. Data from all three replicates presenting the relative change to control are summarized as a heat map in Fig. 5. Most phosphorylated sites with the largest relative increase were identified after induction by NEK2, CK1ε and TTBK2.

In order to assess the impact of excluding phosphopeptides with ambiguous phosphosite localization on quantitative changes, we processed all phosphorylated peptides according to the pipeline #3 (Fig. 3a). Intensities of the phosphorylated clusters using this approach are shown as a heat map in Additional file 2: Figure S2, and illustrate that for 15 clusters analyzed, they match very well the intensities of individual phosphorylated sites presented in Fig. 5. Comparison of results in Fig. 5 and Additional file 2: Figure S2, however, also identified cases where individual phosphorylated sites within the cluster displayed a distinct behavior. Namely, in the cluster T106–S140 the NEK2-induced phosphorylation of T106, S112 and S116 was masked by high intensity constitutive phosphorylation of S125, T133 and S137. In the phosphorylated cluster S202–S209, phosphorylation was decreased for three sites – S202, S203 and S204 – in case of CK1ε and TTBK2 whereas NEK2, on the contrary, induced further phosphorylation of S204. Similarly, for the cluster S598–S612 induced by NEK2, CK1ε, TTBK2 and PKCδ we mapped the activity of NEK2 predominantly to S601, S603 and S605 whereas CK1ε phosphorylated mainly to S611 and S612.

The quantitative data presented in Fig. 5 (accurate positions) and Additional file 2: Figure S2 (clusters) have been combined and analyzed in order to correlate individual kinases with individual phosphorylated sites/clusters. Unbiased cluster analysis (Additional file 3: Figure S3) groups CK1ε and TTBK2, both from the CK1 family, with NEK2, whereas all the remaining kinases form a second group. There are multiple residues that are phosphorylated only by NEK2, which further distinguishes it from CK1ε and TTBK2. Interestingly, CK1ε and TTBK2 behave very similarly and they can be best resolved by the phosphorylation at position S268 that is CK1ε-specific. This is in very good agreement with the site occupancy analysis shown in Fig. 4.

### Phosphorylation map of DVL3: analysis of the phosphorylated clusters

**Fig. 6:**
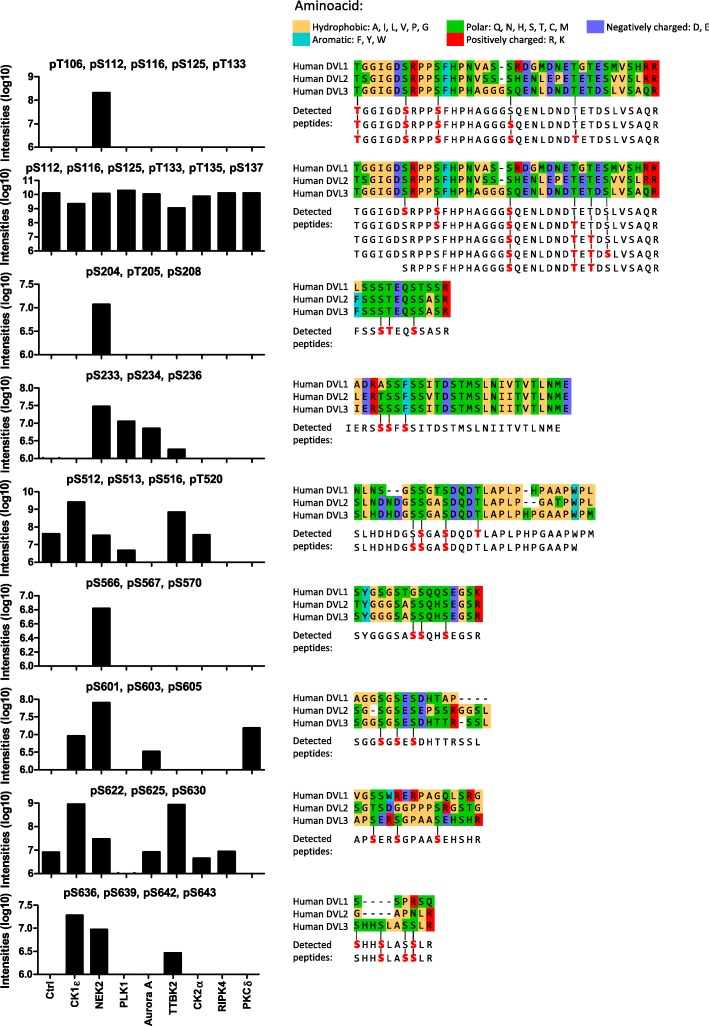
Clusters phosphorylated in more than three sites. The clusters of Ser/Thr where 3 or more phosphorylated sites in one peptide were analyzed (i.e. multiphosphorylated peptides). Graphs indicate total intensities of the multiphosphorylated peptides from all three replicates; signal intensities from pipeline #1 and #3 are merged. Multiple sequence alignment shows the evolutionary conservation of the motif among individual DVL isoforms. All combinations of individual multiphosphorylated peptides detected in the cluster are indicated

The phosphorylated clusters visualized in Additional file 2: Figure S2 can, in principle, represent mixtures of peptides phosphorylated at distinct sites or true multiphosphorylated signatures with possible biological function. To get a better insight, we analyzed in detail the peptides phosphorylated at 3 or more sites. We found 9 peptide families fulfilling these requirements (Fig. 6). Out of these only one cluster – S112, S116, S125, T133, S135, S137 – was not induced by any of the kinases. The remaining 8 clusters were induced by one or several kinases – 3 only by NEK2, 2 mainly by CK1ε and TTBK2 and 3 by NEK2 and one or more additional kinases.

These phosphorylated clusters were detected only in the intrinsically disordered regions of DVL3. The phosphorylated motifs are well conserved and found also in DVL1 and DVL2 except for S622–630 and S636–S643 (Fig. 6, right). This may suggest that the function of multisite phosphorylation of these motifs in the regulation of DVL is also conserved.

### Comparison of individual pipelines and validation by phospho-specific antibodies

**Fig. 7:**
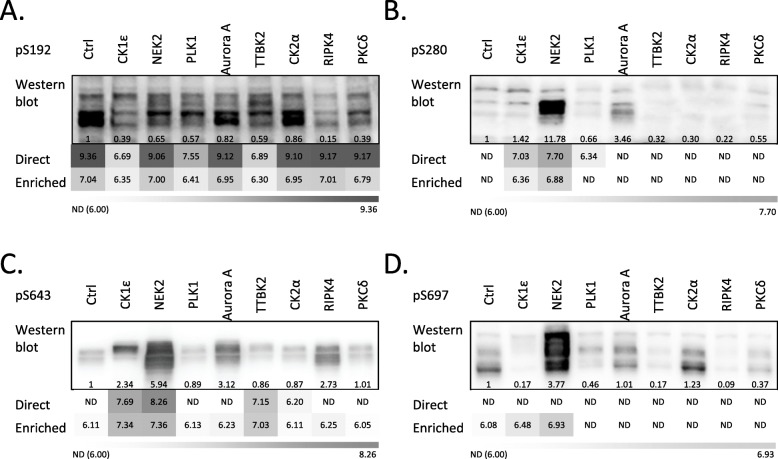
Comparison of individual methods and their validation by phospho-specific antibodies. **a**-**d**. Comparison of methods. Existing phosphorylation-specific antibodies were used to visualize the level of phosphorylation of DVL3 with our kinase panel. HEK293 cells were transfected by the indicated combination of plasmids and analyzed by Western Blotting. Reactivity of individual phosphoantibodies against DVL3 phosphorylated by individual kinases is shown. Western blots are quantified using ImageJ software as absolute values for peak areas of corresponding bands and the intensities were normalized to the control. Mean intensities of the phosphorylated peptides obtained by MS/MS via pipelines #1 and #2 are shown in the shades of grey. Numbers indicate decadic logarithm of the mean peptide intensity. “Not detected” (ND) indicated in white corresponds to the signals below 1 × 106, i.e. 6.0. **a**. anti-pS192 (this study), **b**. anti-pS280, **c**. anti-pS643, **d**. anti-pS697

In our study, we used several sample and data processing pipelines (see Fig. 3). As the last step we compared the individual pipelines (i) between each other and (ii) with several phosphorylation-specific antibodies raised against phosphorylated DVL3 peptides. We probed the samples described in Fig. 2b with antibodies against phosphorylated-S280-DVL3 (pS280) [10], pS643 [20], pS697 [10] and the newly generated anti-pS192. As shown in Fig. 7 a-d that combines Western blots and MS/MS data from pipelines #1 and #2, the signal of phospho-specific antibodies partially matches the changes observed by mass spectrometry. The signal of pS192-DVL3 decreased after CK1ε, PLK1 and TTBK2 co-expression whereas pS280, pS643 and pS697 increased mostly by NEK2. However, some discrepancies were observed, especially for low intensity signals, namely, all three previously validated antibodies pS280, pS643 and pS697 do detect higher phosphorylation after Aurora A co-expression that was not detected by LC-MS/MS. On the other hand, increase in S643 phosphorylation probed for TTBK2 by all MS/MS pipelines was not detected by anti-pS643-DVL3 antibody.

### Phosphoplot – a tool for visualization of complex phosphorylation patterns

**Fig. 8:**
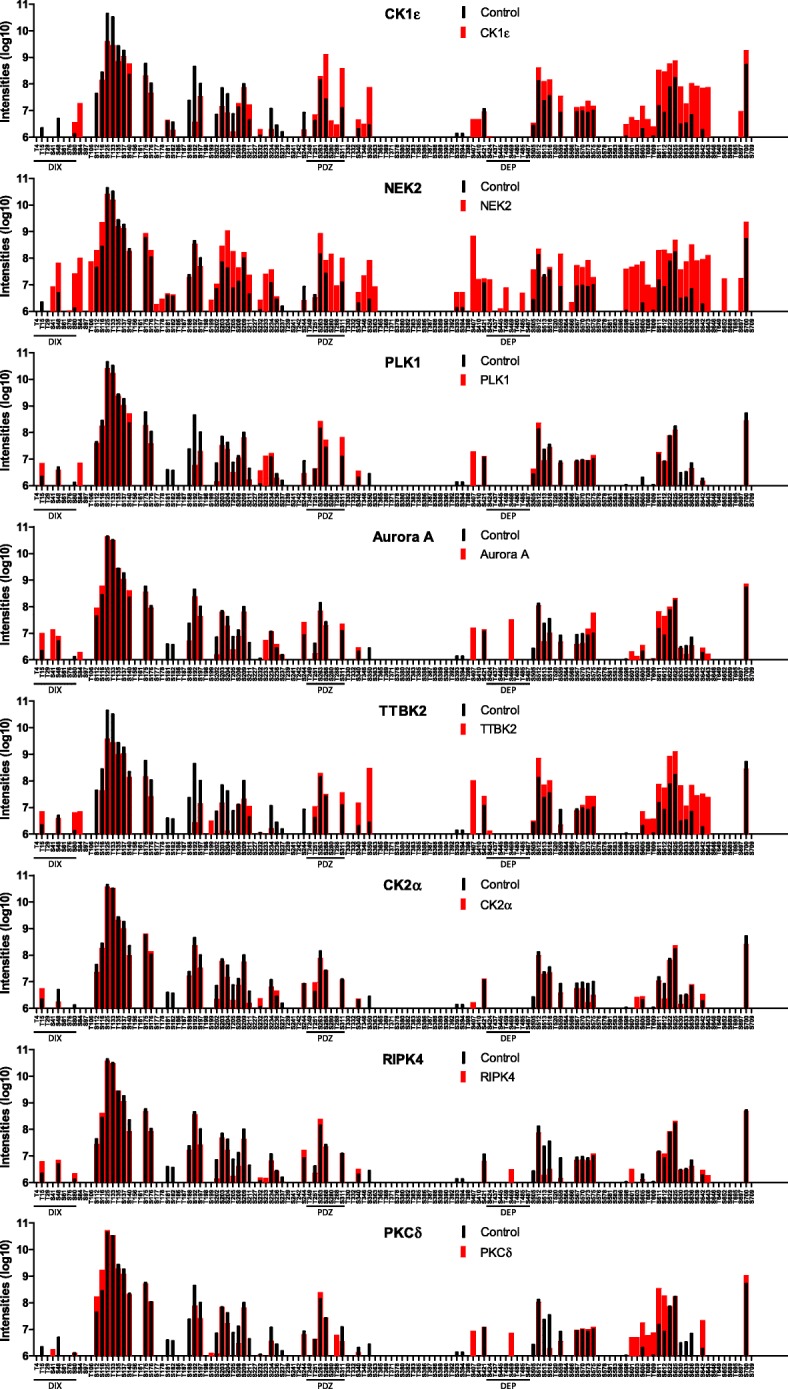
Phosphoplots - phosphorylation barcodes of DVL3 with the individual kinases. Visualization of absolute intensity of phosphorylated peptides corresponding to the individual phosphorylated sites plotted on the primary sequence of DVL3 (only Ser and Thr are shown). Black bars represent a control condition, red bars the intensities in the presence of the kinase. Intensities are plotted on a log10 scale

The heat map data visualization highlights phosphorylation differences in comparison to control but does not provide a full and intuitive picture combining absolute intensities, their changes and position of individual phosphorylated peptides. To address this, we designed a visualization diagram, that we refer to as “phosphoplot”. In the phosphoplot all S/T from the primary sequence of the analyzed protein are shown. The intensities of phosphorylated peptides in the control and experimental conditions are plotted. As such, the phosphoplot combines information on absolute peptide intensities, experimental differences and positional information, including non-phosphorylated sites. The phosphoplots of DVL3 for individual kinases based on data from pipeline #2 are shown in Fig. 8. Phosphoplot inspection identified regions of DVL3 without detected phosphorylation despite being S/T rich – such as T365–T392 between PDZ and DEP domains, and on the contrary, regions between DIX and PDZ domains that are highly constitutively phosphorylated. It also clearly identifies the uniquely phosphorylated sites for each kinase.

### Phosphorylation of S268 and S311 contributes to the activation of the Wnt/β-catenin pathway

**Fig. 9:**
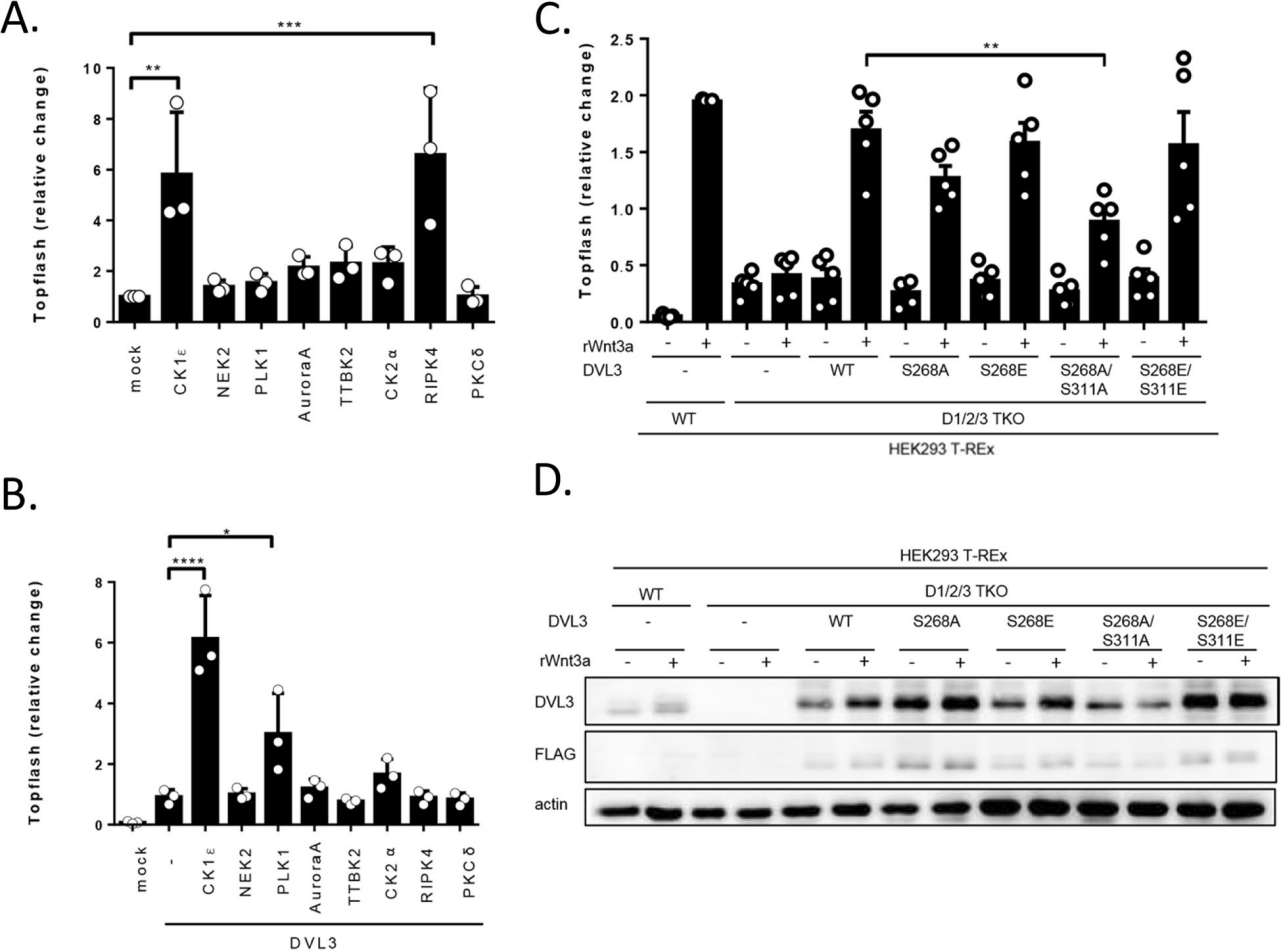
Phosphorylation of S268 and S311 contributes to the activation of the Wnt/b-catenin pathway. **a**, **b**: HEK293 cells were transfected by indicated plasmids together with TopFlash and Renilla reporter plasmids. The ability of individual kinases to induce activation of Wnt/β-catenin pathway either alone (**a**) or in combination with DVL3 (**b**) was analyzed. Mean, SD and individual data points are indicated. Statistical differences were tested by One-way ANOVA and Tukey’s post test (* *p* < 0.05, ** *p* < 0.01, *** *p* < 0.001, **** *p* < 0.0001). **c**, **d**: Rescue experiments with DVL3 S268/S311 mutants. WT and D1/2/3 TKO HEK293 T-Rex cells were transfected as indicated and treated according to the scheme with 80 ng/ml recombinant human Wnt3a (rWnt3a). All samples were treated with 0.1 μM LGK974 inhibitor and 250 ng/ml R-SPONDIN1 and analyzed by TopFlash assay (**c**). Mean, SD and individual data points are indicated. Statistical differences were tested by paired t-test (* *p* < 0.05, ** *p* < 0.01). Samples were also used for WB analysis (**d**)

To associate any phosphorylation patterns with the function of DVL we compared individual kinases in their capacity to induce Wnt/β-catenin-dependent transcription analyzed by the TopFlash reporter assay. In the absence of exogenous DVL3 only CK1ε and RIPK4 significantly activated TCF/LEF-driven transcription (Fig. 9a). When DVL3 was co-expressed, only CK1ε, and to a lesser extent PLK1 (non-significant trend was observed also for CK2α), could synergize with DVL3 to promote reporter activation (Fig. 9b). We could largely reproduce the reported effects: the capacity of CK1ε and RIPK4 to activate TopFlash reporter [19, 48], and the synergistic behavior of DVL3 and CK1ε in the TopFlash reporter [16]. Although, it has been proposed that RIPK4 activates Wnt/β-catenin also via phosphorylation of DVL [19] we have not observed any synergy with DVL3 which suggests that RIPK4 may act via other proteins in the Wnt/β-catenin pathway.

CK1ε stands unique among other kinases in its ability to efficiently induce Wnt/β-catenin downstream signaling in synergy with DVL3. We have thus analyzed the data reported in Figs. 4, 5 and Additional file 3: Figure S3 and identified S268 and S311 as candidate phosphorylation sites associated with the expression of CK1ε and activation of TopFlash reporter assay. In order to test to what extent phosphorylation of S268 and S311 participates in the activation of Wnt/β-catenin downstream signaling we mutated S268 alone or in combination with S311 to alanine and glutamic acid (S268A, S268E, S268A/S311A, S268E/S311E) and tested these DVL3 mutants for their capacity to rescue Wnt-3a signaling in DVL1/DVL2/DVL3-triple null HEK293 T-REx cells (D1/2/3 TKO cells) [47]. As shown in Fig. 9 C/D, DVL3 S268A and especially S268A/S311A are significantly less efficient in their ability to rescue Wnt-3a-induced activity. This data suggests that phosphorylation of DVL3 at S268 and S311 by CK1ε is one of the essential steps in the Wnt-3a-induced activation of β-catenin signaling.

### Phosphorylation of S630-S643 mediates even localization of DVL3

**Fig. 10:**
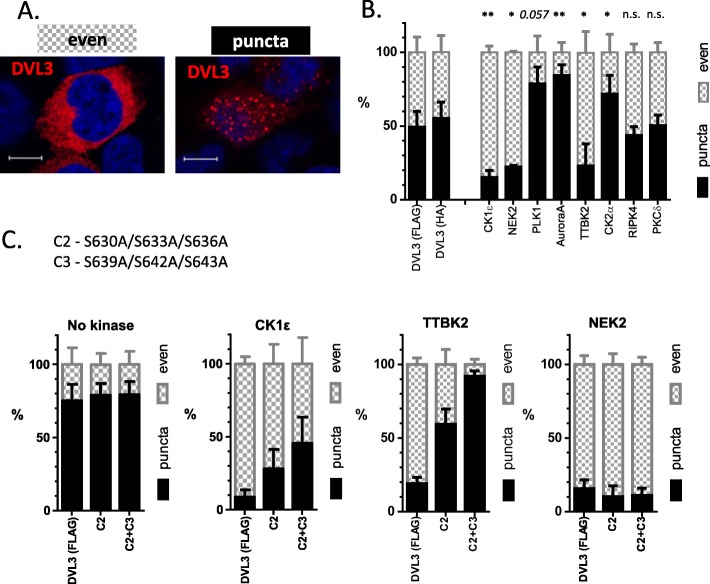
Phosphorylation of S630-S643 promotes even localization of DVL3. **a**: HEK293 cells were transfected in the indicated combinations and the subcellular localization of DVL3 was assessed by immunocytochemistry. DVL3 was localized in two typical patterns – either in cytoplasmic puncta or evenly dispersed in the cytoplasm (upper panel). Scale bar, 7.5 μm. **b**: The effects of individual kinases on DVL3 localization is shown in the bottom panel (HA-DVL3 was used for PLK1 and TTBK2, FLAG-DVL3 for the rest of kinases). **c**: Phosphorylation patterns associated with the even localization of DVL3 were analyzed by mutation of cluster of serine residues to alanine. Statistical data represent mean + SD from three independent experiments (*N* = 3× 200 cells). Statistical significance was confirmed by the comparison of the corresponding control (DVL3-FLAG or DVL3-HA without kinase) and DVL3 with individual kinases by One-way ANOVA and Tukey’s post test (* *p* < 0.05, ** *p* < 0.01, n.s. - not significant)

Subcellular localization of DVL3 is dynamically regulated. Overexpressed DVL3, similarly to other DVL proteins, is localized in the “DVL punctae”; protein assemblies kept together via polymerization of DVL DIX domains [49]. These assemblies are dynamic and can be more compact (visible as puncta) or dissolved, resulting in the “even” distribution of DVL3 (examples of these two DVL states are presented in Fig. 10a). As shown in Fig. 10b, bottom panel, only three kinases – CK1ε, NEK2 and TTBK2 – were capable to promote even localization of DVL3.

In order to identify phosphorylation patterns associated with the even localization of DVL3 we analyzed common phosphorylation events induced by CK1ε, TTBK2 and NEK2. A cluster of the regularly spaced serines S630-S633-S636-S639-S642-S643 was the most obvious candidate for such function (see Figs. 4, 5 and 6). Mutation of these residues to alanine (A) prevented the ability of CK1ε (Fig. 10c) to induce the even distribution of DVL3, in line with an earlier report [23]. Similar and even stronger effect was observed for TTBK2 suggesting that TTBK2 acts via a similar mechanism as CK1ε and that phosphorylation of S630-S643 is essential for TTBK2-induced even localization of DVL3. Surprisingly, NEK2 efficiently promoted even localization of DVL3 in all DVL3 mutants (Fig. 10c, right). This suggested that although NEK2 strongly phosphorylates S630-S643 cluster, it is able to overcome the requirement for S630-S643 phosphorylation and efficiently induce even localization of DVL3 also via a different mechanism.

### Phosphorylation of S263 and S280 by NEK2 induces open conformation of DVL3

**Fig. 11:**
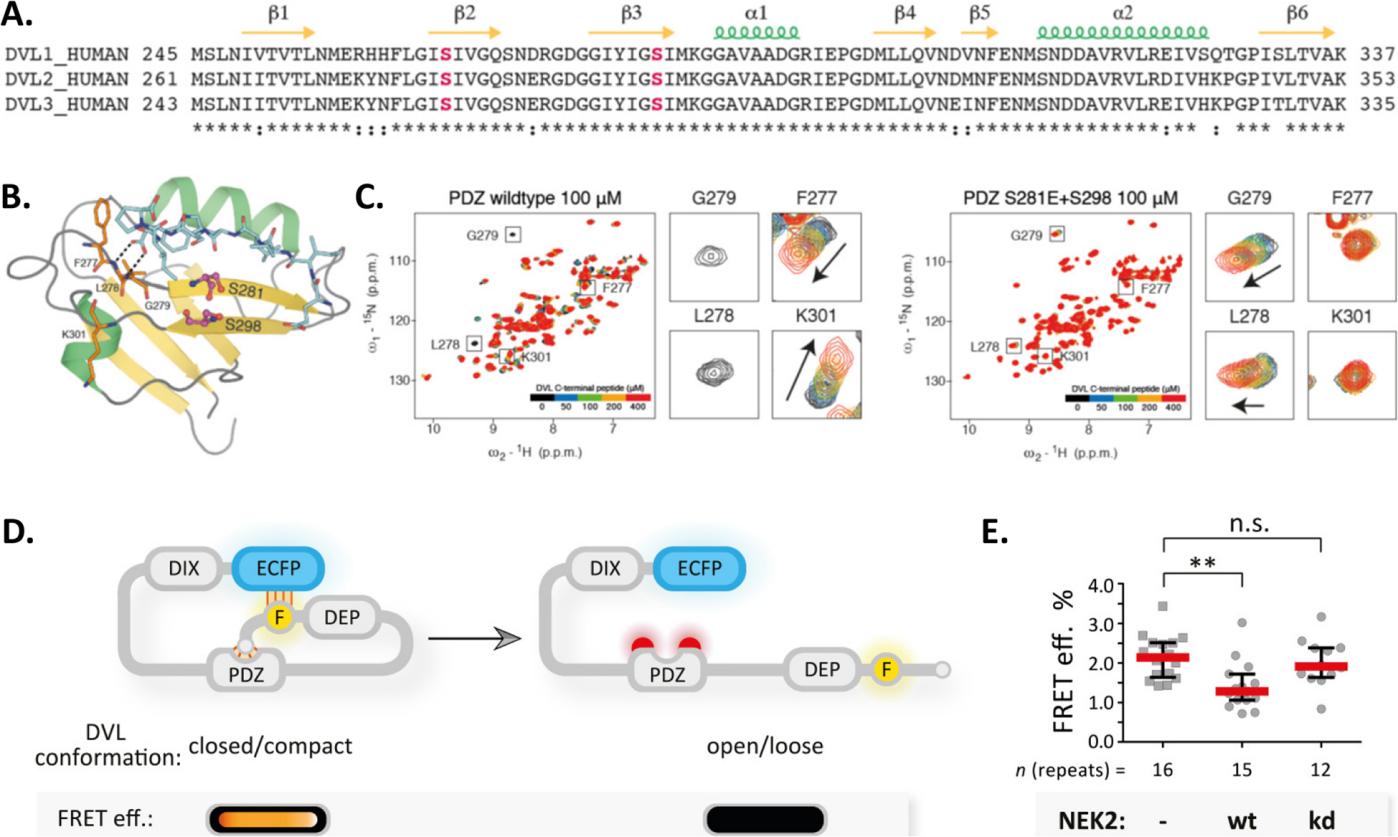
NEK2 phosphorylation in PDZ domain promotes open conformation of DVL3. **a**: PDZ sequence alignment of the three human DVL isoforms. The conserved serine residues phosphorylated specifically by NEK2 are highlighted in magenta. **b**: Crystal structure of DVL2 PDZ domain bound to internal ligand (PDB: 3CC0). The two serines prone to NEK2 phosphorylation are shown in magenta, the bound peptide in cyan, and PDZ residues of the binding loop or close to the binding loop in orange. **c**: NMR titrations of DVL3 C-terminal peptide to DVL2 wildtype PDZ or S281E/S298E phosphomimicking mutant. Magnified insets show the qualitative differences in peptide binding for PDZ residues annotated in (**b**). **d**: Schematic depiction of the FRET efficiency experiment of the ECFP DVL3 FlAsH III construct. In the default conformation, the DVL molecule is less phosphorylated and closed/compact, which is reflected in the close proximity of the FRET pair (ECFP and FlAsH (F) tags) – leading to the high intramolecular FRET efficiency (depicted as orange dash lines). In the presence of the active kinase (e.g. NEK2), DVL is phosphorylated in the PDZ domain, which promotes the open/loose conformation by the disruption of the C-terminus/PDZ domain interaction. This is reflected in the high proximity of ECFP and F tags thus leading to the low intramolecular FRET efficiency (no orange dash lines). **e**: HEK293 cells were transfected in the indicated combinations and the intramolecular FRET efficiency of the ECFP-DVL3 FlAsH III construct (schematically depicted in D) was measured. The data represent median ± interquartile range from three independent experiments (numbers of analyzed cells are indicated below). Statistical significance was confirmed by One-way ANOVA and Tukey’s post test (** *p* < 0.01, n.s. - not significant). FRET eff. Stands for Förster-Resonance-Energy-Transfer efficiency, ECFP for Enhanced Cyan Fluorescent Protein, F for FlAsH tag, and kd for kinase dead

We have recently shown a correlation between the subcellular localization of DVL3 and DVL3 conformation [24]. DVL3 forms a closed conformation via an intramolecular interaction between the C-terminus and the PDZ domain [50]. To test whether a similar mechanism can overcome the requirement for phosphorylation of S630-S643 cluster by NEK2 we identified the phosphorylation sites in the PDZ domain uniquely induced by NEK2. S263 and S280 fit the criteria as the candidate sites for functional validation (see Fig. 4 and Fig. 5). In order to address the possible role of DVL3 phosphorylation-driven regulation involving S263 and S280 in vitro, we performed functional characterization by NMR. For this purpose, we exploited the well-characterized PDZ domain of human DVL2 (aa 265–361) [51], in which S281 and S298 correspond to S263 and S280 of human DVL3. As seen in the crystal structure of the complex between DVL2 PDZ and an internal peptide ligand [52] the fully conserved S281 and S298 (Fig. 11a) reside at adjacent strands of the PDZ fold in close proximity to the peptide binding groove (Fig. 11b). From the NMR fingerprint spectra, we concluded that the phosphorylation-mimicking substitutions (S281E/S298E) did not affect the integrity of the PDZ fold (Additional file 4: Figure S4). Next, we assessed PDZ binding to a C-terminal peptide ligand derived from DVL3 (aa 698–716) by NMR titrations [24]. The residues in the binding loop region (L278 and G279) that serve as the H-bonding donors to the carboxyl sidechain of the peptide Asp sidechain undergo severe line broadening in wildtype PDZ, whereas in the phosphomimicking mutant experience fast exchange perturbation (Fig. 11c). Several other peaks (e.g. F277 and K301) that are largely perturbed in the course of the titration in wildtype remain unaffected in mutant (Fig. 11c). The qualitative analysis of the titration experiments indicates that the phosphomimicking S281E/S298E PDZ is insufficient in peptide binding as compared to wt PDZ (Fig. 11c). This suggests that phosphorylation of S263 and S280 (corresponding to S281 and S298 in DVL2) attenuates the capacity of PDZ domain to bind the C-terminus of DVL3 and therefore leads to open conformation of DVL3.

To directly test whether NEK2 indeed induces open conformation of DVL3 we employed the DVL3 FRET sensors [24]. Specifically, the ECFP-DVL3 FlAsH III sensor (schematized in Fig. 11d) was transfected in HEK293 cells either alone or in the presence of wild type (WT) and kinase dead (KD) NEK2 and subsequently, the intramolecular FRET efficiency was quantified. As shown in Fig. 11e, only WT (but not KD) NEK2 was able to lower the intramolecular FRET efficiency that depends on the proximity of the ECFP and FlAsH (F) tag. This result suggests that the kinase activity of NEK2 (and not the presence of NEK2 itself as demonstrated by the KD variant) promotes the open conformation of DVL3. In summary with other data, we propose that NEK2-induced phosphorylation of S263 and S280 in human DVL3 prevents the interaction between DVL’s C-terminus and PDZ domain, thus keeping DVL in the open conformation and more evenly distributed.

## Discussion

Our study provides the first comprehensive description of DVL3 phosphorylation by most of the described DVL S/T kinases. Given the high sequence conservation of DVL3 and other DVL proteins it is an important reference point for the interpretation of published data as well as a benchmark for forthcoming studies focused on the regulation of DVL (and other proteins) function by kinases and phosphatases.

In this study we have identified TTBK2 and Aurora A as novel DVL kinases. For TTBK2 we propose a possible negative role in Wnt/β-catenin signaling. TTBK2 is found to basal bodies or distal appendages, respectively. Indeed, DVL has been associated with centrosome and basal bodies [6, 10, 53]. However, it does not seem to localize to distal appendages of mother centriole, where TTBK2 is recruited by CEP164 to trigger ciliogenesis [34, 40, 42–45]. It remains to be determined what is the possible interplay between TTBK2 activity and DVL3 phosphorylation by Aurora A, NEK2 and PLK1 and how these kinases together regulate DVL localization and functions in the individual phases of the cell cycle.

The parallel application of several pipelines for sample preparation and data analysis, in particular the newly introduced site occupancy analysis, demonstrated the potential to detect phosphorylation events that are biologically relevant. This approach identified S268 as signature site for CK1ε, S280 as a signature site for NEK2 and S633–S643 cluster as a signature for CK1ε, NEK2 and TTBK2. All these phosphorylation changes have been validated by additional, more demanding proteomic pipelines and functional analysis.

The data collected by various approaches shown in Figs. 4, 5, 6, 7, provide a global view of DVL3 phosphorylation induced by kinases that were shown to control distinct functions of DVL. We can clearly observe codes of phosphorylated S/T in the structured domains – most typically in the PDZ domain at the fully conserved S263, S268, S280 and S311, and to a lesser extent also in the DIX and DEP domains. On the other hand, in the largely intrinsically disordered regions (IDRs) between the domains, we have detected conserved (see Fig. 6) multiphosphorylated sequences with specific phosphorylation patterns. Most intrinsically disordered proteins or regions are very S/T rich because these aa (together with A, R, G, Q, P, E and K) are disorder-promoting. IDR-rich proteins (such as DVL) are essential components of multiple signal transduction pathways [54] and complex multiphosphorylation as shown for DVL can represent a shared and universal mechanism for the regulation of their function.

It is behind the scope of this study to test the functional consequences of all phosphorylation events. However, several interesting candidates for further analysis have been identified. For example, a striking phosphorylation pattern has been observed for two highly conserved regions between DIX and DEP domains – corresponding to S112–S140 and S188–S197. For the second cluster we were able to confirm the proteomic data by a phosphorylation-specific antibody targeted against pS192-DVL3. These sites showed high level of basal phosphorylation in the absence of any exogenously co-expressed kinase. Interestingly, expression of CK1ε (and to a lesser extent TTBK2 and PLK1) dramatically reduced phosphorylation of these motifs. This suggests that there is a so far unidentified endogenous kinase that very efficiently phosphorylates these regions, and (ii) that the binding and/or phosphorylation by CK1ε and TTBK2 interferes with this process or perhaps promotes removal of these phospho-moieties by activation of specific phosphatase(s). So far, only protein phosphatase 2A has been shown to have a positive function in the Wnt/β-catenin signaling upstream of DVL [55, 56], which makes it an ideal candidate for such function.

Our functional analysis of PTMs that control subcellular localization of DVL3 showed that phosphorylation in the domains (namely PDZ) and in the IDRs can lead to the same outcome. CK1ε, NEK2 and TTBK2, were the only kinases that could trigger the even distribution of DVL3 in the cytoplasm, in line with earlier reports [10, 12, 13] [20]. Interestingly, phosphorylation of the cluster S630–S643 was essential for the ability of CK1ε and TTBK2 (but not of NEK2) to trigger even localization of DVL3. It appears that NEK2 uses a different mechanism dependent on the phosphorylation of S263 and S280 in the PDZ domain. Therefore, DVL C-terminus cannot bind to PDZ, which in turn stabilizes DVL3 in the open conformation. Importantly, despite the fact that the open conformation of DVL3 correlates with the even subcellular localization and more efficient recruitment to FZD receptors [24], it does not necessarily translate into the activation of the Wnt/β-catenin signaling. Our data are rather compatible with the hypothesis that phosphorylation opens an auto-inhibited DVL conformation and then the kinase-specific barcode dictates the biological outcome. Phosphorylated S268 and S311 can be part of such barcode required for CK1ε-induced activation of the Wnt/β-catenin signaling [11, 13] whereas phosphorylated S263 and S280 are barcode components essential for the NEK2-controlled functions of DVL3 in the centrosome [10].

## Conclusions

In summary, our study identified unique DVL3 phosphorylation barcodes associated with individual DVL kinases and DVL functions. Our data represent an important reference point and a toolbox for further analysis of DVL as exemplified by the functional analysis of the phosphorylation in the PDZ domain. From the more general point of view, our data pinpoint the importance of functional synergy between phosphorylation in the structured domains and in the unstructured IDRs that together dictate the biological outcome.

## Supplementary information

**Additional file 1: Figure S1.** SDS-PAGE gels from three independent experiments. FLAG-DVL3 was overexpressed in HEK293, with or without the studied kinase, immunoprecipitated using the anti-FLAG antibody, separated on SDS-PAGE and stained with Coomassie Brilliant Blue. In red boxes are parts that were cut out.

**Additional file 2: Figure S2.** Phosphorylated clusters map of DVL3. All identified phosphorylated clusters obtained from the pipeline #3 are visualized as a heatmap. Mean absolute intensities of phosphorylation clusters in the control (DVL3 without exogenous kinase; six replicates) are expressed in the shades of blue. Numbers indicate decadic logarithm of the mean of 6 control samples. Nine columns represent heat map of relative change of phosphorylated peptide intensities (in log10 scale) obtained for individual kinases (relative to control). Numbers in the heat map fields (0, 1, 2, 3) indicate the number of experimental replicates with the positive identification of the given phosphorylated site.

**Additional file 3: Figure S3.** Cluster analysis of the individual phosphorylated sites/clusters and individual kinases. Mean phosphorylated peptide intensities (in log10 scale) standardized to control are used in the circular plot. Peptides expressed more intensively compared to control are in the shades of green, whereas less expressed peptides are in red. Dendrograms both for the kinases and the phosphosites are shown.

**Additional file 4: Figure S4.** NMR comparison of DVL2 PDZ wild-type and phosphorylation-mimicking mutant S281E + S298E. A. 1H, 15 N HSQC spectra of PDZ wild-type (blue) and phosphorylation-mimicking mutant S281E + S298E (red). All amide frequencies have been assigned unambiguously using 4D spectra. The e cross-peaks of wild-type and corresponding glutamic acids cross-peaks of the mutant are marked. B. Weighted chemical shift differences between PDZ wild-type and S281E + S298E mutant versus the protein sequence.

**Additional file 5: Table S1.** Intensities of the individual phosphorylated sites (raw data). Mean phosphorylated peptide intensities (in log10 scale) obtained for individual sample from direct (pipeline #1) and enriched analysis processed (pipeline #2) are expressed in the shades of blue. ND corresponds to not detected signals or signals with intensity below 1 × 10^6^.

**Additional file 6: Table S2.** Sequence coverage of DVL3 in the individual experiments. Sequence coverage of DVL3 obtained for individual kinases in each replicate, in individual sample (sum of all three replicates) and throughout the experiment.

## Data Availability

Proteomic data are available via PRIDE.

## Abbreviations

AB: Ammonium bicarbonate
CK: Casein kinase
DAPI: 4′,6-diamidino-2-phenylindole
DMEM: Dulbecco’s modified eagle’s medium
DTT: Dithiotreitol
DVL: Dishevelled protein in human
ECFP: Enhanced cyan fluorescent protein
EDT: 1,2-ethanedithiol
FCS: Fetal calf serum
FlAsH: Fluorescein arsenical hairpin binder
GSK: Glycogen synthase kinase
HBSS: Hank’s balanced salt solution
HCD: Higher-energy collisional dissociation
IAA: Iodoacetamide
IDR: Intrinsically disordered region
NEK2: Nima-related kinase 2
PEG: Polyethyleneglycol
PEI: Polyethylenimine
PFA: Paraformaldehyde
PKCδ: Protein kinase C δ
PLK1: Polo-like kinase 1
RIPK4: Receptor-interacting protein kinase 4
TCF/LEF: T-cell factor/lymphoid enhancer-binding factor
TTBK2: Tau tubulin kinase 2
