# Supplementary material for: Comparative phosphorylation map of Dishevelled 3 links phospho-signatures to biological outputs
Source: Cell Commun Signal. 2019 Dec 23;17:170. doi: 10.1186/s12964-019-0470-z (PMC6927192; doi:10.1186/s12964-019-0470-z)
Supplement: Supplementary file 2 [file 12964_2019_470_MOESM3_ESM.docx]

Additional file 2: Figure S2

DVL3	Mean intensity of control	Relative change of phosphopeptide intensities								
		Control	CK1ε	NEK2	PLK1	Aurora A	TTBK2	CK2α	RIPK4	PKCδ
S76, S80, S84	6.17	2	3	3	3	2	3	2	2	1
T106–S140	10.83	3	3	3	3	3	3	3	3	3
S175, S176, S177	8.77	3	3	3	3	3	3	3	3	3
T178–S182	6.57	2	1	2	1	1	1	1	0	0
S188, S192, S197	8.16	3	3	3	3	3	3	3	3	3
S202–S209	8.28	3	3	3	3	3	3	3	3	3
S232–S237	7.26	3	2	3	3	3	3	3	3	3
T251, Y257	6.36	1	1	1	1	1	1	1	1	1
S393, S394	6.24	1	0	1	0	0	0	0	0	0
S407, S410	6.06	1	1	1	1	1	1	1	1	1
S505–S516	7.84	3	3	3	2	3	3	2	2	2
S559–S575	7.63	3	3	3	3	3	3	3	3	3
S598–S612	7.13	3	3	3	2	3	3	3	2	3
S622, S625, S630	8.17	3	3	3	3	3	3	3	3	3
S633–S643	6.88	3	3	3	3	3	3	3	3	2

ND (6.00) 11.00 -1.50 2.00
