# Supplementary material for: Comparative phosphorylation map of Dishevelled 3 links phospho-signatures to biological outputs
Source: Cell Commun Signal. 2019 Dec 23;17:170. doi: 10.1186/s12964-019-0470-z (PMC6927192; doi:10.1186/s12964-019-0470-z)

Additional file 4: Figure S4

A.

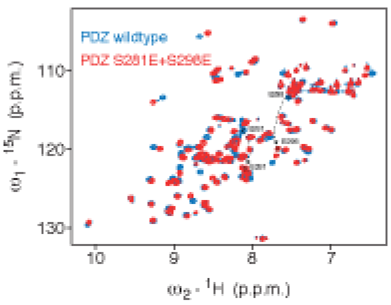

B.

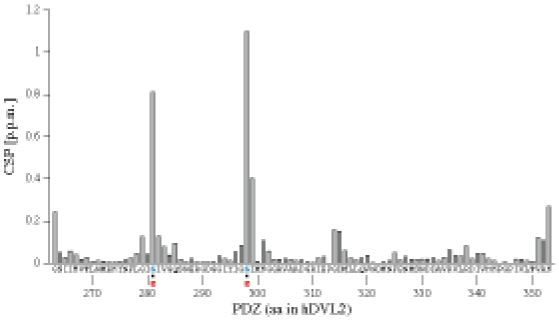

Supplement: Supplementary file 4 [file 12964_2019_470_MOESM5_ESM.docx]
