# Supplementary material for: Comparative phosphorylation map of Dishevelled 3 links phospho-signatures to biological outputs
Source: Cell Commun Signal. 2019 Dec 23;17:170. doi: 10.1186/s12964-019-0470-z (PMC6927192; doi:10.1186/s12964-019-0470-z)
Supplement: Supplementary file 5 [file 12964_2019_470_MOESM6_ESM.docx]

Additional file 5: Table S1

Phosphorylated sites	Control 1		CK1α		NEK2		PLK1		Aurora A		Control 2		TTBK2		CK2α		RIPK4		PKCδ	
	Direct	Enriched	Direct	Enriched	Direct	Enriched	Direct	Enriched	Direct	Enriched	Direct	Enriched	Direct	Enriched	Direct	Enriched	Direct	Enriched	Direct	Enriched
	ND (6.00)																			11.00
T15	7.85	6.20	8.00	6.15	7.96	ND	8.15	6.44	7.89	6.49	7.95	6.32	7.87	6.44	7.93	6.41	7.72	6.42	7.95	ND
S41	ND	ND	ND	ND	7.38	6.72	ND	ND	7.16	6.67	ND	ND	ND	ND	ND	ND	6.39	ND	ND	6.24
S48	ND	6.65	ND	ND	ND	7.61	ND	6.36	ND	6.46	ND	6.42	ND	6.47	ND	6.24	ND	6.44	ND	ND
S61	ND	ND	ND	ND	ND	ND	ND	ND	ND	ND	ND	ND	ND	6.12	ND	ND	ND	ND	ND	ND
S76	ND	ND	ND	ND	ND	6.17	ND	ND	ND	ND	ND	ND	ND	ND	ND	ND	ND	ND	ND	ND
S80	ND	6.23	6.23	6.46	6.92	6.97	6.36	6.09	6.44	6.13	6.35	6.12	6.40	6.65	ND	6.10	ND	6.28	ND	6.19
S84	6.42	ND	7.33	7.21	8.16	7.75	7.12	6.84	6.35	6.28	ND	ND	6.36	6.77	6.10	ND	ND	ND	ND	ND
T106	ND	ND	ND	ND	ND	7.67	ND	ND	ND	ND	ND	ND	ND	ND	ND	ND	ND	ND	ND	ND
S112	ND	6.71	ND	ND	ND	7.62	ND	6.69	ND	6.81	ND	6.69	ND	ND	ND	6.61	ND	6.64	ND	6.90
S116	ND	8.42	ND	8.15	ND	9.20	ND	8.15	ND	8.77	ND	8.31	ND	7.59	ND	8.21	ND	8.55	ND	8.95
S125	9.68	10.64	8.95	9.56	9.77	10.33	9.81	10.33	9.55	10.60	9.77	10.57	8.70	9.57	9.54	10.56	9.73	10.56	9.66	10.62
T133	9.53	10.35	8.83	9.27	9.45	9.96	9.75	10.11	9.42	10.32	9.63	10.28	8.60	9.31	9.43	10.32	9.60	10.29	9.55	10.34
T135	9.29	9.43	8.75	7.95	9.31	9.17	9.58	9.36	9.17	9.43	9.39	9.36	8.40	7.97	9.21	9.12	9.38	9.42	9.35	9.28
S137	6.36	9.23	6.20	8.97	6.69	9.07	6.30	9.00	ND	9.03	6.43	9.18	ND	8.80	6.38	8.96	6.09	9.03	ND	9.07
S140	ND	7.75	ND	7.78	ND	7.62	ND	7.88	ND	7.78	ND	7.55	ND	7.51	ND	7.43	ND	7.34	ND	7.51
S175	ND	8.80	ND	8.17	ND	8.76	ND	8.11	ND	8.47	ND	8.62	ND	8.13	ND	8.68	ND	8.61	ND	8.66
S176	8.14	7.46	7.20	7.18	8.19	7.58	7.65	7.16	7.94	7.41	7.87	7.46	6.88	7.04	7.91	7.53	7.58	7.40	8.01	7.47
S177	ND	6.10	ND	ND	ND	6.25	ND	ND	ND	ND	ND	ND	ND	ND	ND	ND	ND	ND	ND	ND
T178	ND	ND	ND	ND	ND	6.31	ND	ND	ND	ND	ND	ND	ND	ND	ND	ND	ND	ND	ND	ND
S181	ND	6.31	ND	6.37	ND	6.45	ND	ND	ND	ND	ND	6.39	ND	ND	ND	6.08	ND	ND	ND	ND
S182	ND	6.31	ND	6.25	ND	6.54	ND	6.09	ND	6.10	ND	6.53	ND	6.10	ND	6.08	ND	ND	ND	ND
S188	ND	6.64	ND	6.16	ND	6.59	ND	ND	ND	6.40	ND	6.58	ND	ND	ND	6.56	ND	6.57	ND	ND
S192	9.33	7.05	6.69	6.35	9.06	7.00	7.55	6.41	9.12	6.95	9.39	7.03	6.89	6.30	9.10	6.95	9.17	7.01	9.17	6.79
S197	7.16	7.95	6.18	7.08	6.93	7.62	ND	7.21	7.15	7.59	7.38	7.75	ND	6.98	6.55	7.30	ND	7.38	6.73	7.32
S199	ND	ND	ND	ND	ND	6.30	ND	ND	ND	ND	ND	ND	ND	6.32	ND	ND	ND	ND	ND	6.20
S202	ND	6.65	ND	ND	ND	6.66	ND	6.18	ND	6.22	ND	6.58	ND	6.08	ND	6.34	ND	6.20	ND	6.17
S203	8.05	7.47	6.53	6.77	8.78	8.11	7.88	7.30	7.86	7.35	8.17	7.43	6.33	6.76	8.15	7.29	8.06	7.21	7.45	7.35
S204	7.46	7.58	6.44	6.75	9.16	9.01	7.01	7.34	6.81	7.26	7.04	7.50	ND	6.15	7.16	7.15	6.85	7.08	6.80	7.17
T205	ND	6.46	ND	6.23	7.50	7.27	ND	6.33	ND	6.29	ND	6.44	ND	ND	ND	6.26	ND	6.24	ND	ND
S208	ND	7.08	ND	7.18	ND	7.55	ND	6.97	ND	6.86	ND	7.00	ND	6.91	ND	6.70	ND	6.58	ND	6.43
S209	7.97	7.72	7.30	7.72	8.03	7.87	7.83	7.67	7.79	7.59	7.76	7.73	6.77	7.30	7.63	7.61	7.40	7.32	7.07	7.50
S211	ND	6.38	ND	7.04	ND	7.02	ND	6.23	ND	6.10	ND	6.36	ND	6.65	ND	6.22	ND	ND	ND	6.12
S232	ND	6.19	ND	6.25	ND	6.30	ND	6.35	ND	6.10	ND	6.16	ND	6.17	ND	6.28	ND	6.21	ND	6.17
S233	ND	6.11	ND	ND	ND	7.32	ND	6.85	ND	6.60	ND	6.13	ND	6.16	ND	6.13	ND	6.21	ND	ND
S234	ND	6.56	ND	6.26	ND	7.46	ND	7.04	ND	6.75	ND	6.44	ND	6.23	ND	6.43	ND	6.43	ND	6.35
S236	ND	6.27	ND	ND	ND	6.35	ND	6.26	ND	6.50	ND	6.42	ND	ND	ND	6.48	ND	6.39	ND	6.10
S237	ND	6.27	ND	ND	ND	ND	ND	ND	ND	6.22	ND	6.13	ND	ND	ND	6.13	ND	6.17	ND	ND
S244	ND	6.52	ND	6.25	ND	6.30	ND	6.31	ND	6.63	ND	6.39	ND	6.14	ND	6.47	ND	6.56	ND	6.43
T251	ND	6.32	ND	6.44	ND	6.33	ND	6.37	ND	6.24	ND	6.40	ND	6.50	ND	6.48	ND	6.28	ND	6.37
Y257	ND	6.32	ND	6.44	ND	6.33	ND	6.37	ND	6.24	ND	6.40	ND	6.50	ND	6.48	ND	6.28	ND	6.37
S263	ND	6.92	6.88	8.10	9.03	8.82	8.54	8.38	6.50	7.30	6.20	6.94	6.34	7.96	ND	6.93	ND	7.10	ND	7.39
S268	6.95	7.35	8.93	9.05	7.06	7.70	7.23	7.67	6.68	7.19	6.68	7.30	6.56	7.40	6.19	7.26	6.68	7.28	6.57	7.31
S280	ND	ND	7.03	6.36	7.70	6.88	6.34	ND	ND	ND	ND	ND	ND	ND	ND	ND	ND	ND	ND	ND
T288	ND	ND	ND	6.31	ND	6.59	ND	ND	ND	ND	ND	ND	ND	ND	ND	ND	ND	ND	ND	6.42
S311	6.38	6.52	7.81	7.02	6.71	6.83	6.65	6.76	6.39	6.61	ND	6.52	6.81	6.68	6.35	6.51	ND	6.52	ND	6.34
S340	6.07	6.34	6.81	6.63	7.13	6.63	7.08	6.54	6.49	6.41	6.36	6.19	7.56	7.17	6.35	6.32	7.01	6.47	6.27	6.18
T346	ND	ND	ND	6.42	ND	6.96	ND	ND	ND	ND	ND	ND	ND	ND	ND	ND	ND	ND	ND	ND
S350	6.70	6.27	7.78	7.36	7.79	7.37	ND	ND	6.59	ND	6.67	6.46	8.04	8.42	6.77	ND	ND	ND	ND	ND
S363	ND	ND	ND	ND	ND	6.47	ND	ND	ND	ND	ND	ND	ND	ND	ND	ND	ND	ND	ND	ND
S393	ND	6.24	ND	ND	ND	6.40	ND	ND	ND	ND	ND	6.15	ND	ND	ND	ND	ND	ND	ND	ND
S394	ND	6.24	ND	ND	ND	6.40	ND	ND	ND	ND	ND	6.15	ND	ND	ND	ND	ND	ND	ND	ND
S407	6.46	ND	ND	6.38	8.73	7.10	7.80	6.58	6.68	6.56	6.42	6.11	8.31	6.83	ND	6.23	ND	6.08	6.42	6.47
S410	ND	ND	ND	6.38	ND	6.56	ND	ND	ND	ND	ND	ND	ND	ND	ND	ND	ND	ND	ND	ND
S421	6.36	7.02	ND	6.75	6.37	7.21	6.54	7.04	6.38	7.00	6.34	6.80	6.54	7.39	6.23	7.10	6.42	6.75	ND	7.06
S424	ND	ND	ND	6.17	6.44	6.55	ND	ND	6.27	ND	6.20	6.11	6.21	6.20	ND	ND	ND	ND	ND	ND
S445	ND	ND	ND	ND	6.42	6.19	ND	ND	ND	ND	ND	ND	ND	ND	ND	ND	ND	ND	ND	ND
T459	ND	ND	ND	6.01	ND	6.64	ND	ND	ND	ND	ND	ND	ND	ND	ND	ND	ND	ND	ND	ND
S469	6.50	6.07	ND	ND	6.93	ND	6.43	6.14	6.77	6.67	6.40	6.11	6.41	6.13	6.45	6.14	6.56	6.32	6.47	6.45
T485	ND	ND	ND	ND	6.61	6.39	ND	ND	ND	ND	ND	ND	ND	ND	ND	ND	ND	ND	ND	ND
S505	ND	6.50	6.90	6.47	7.57	7.32	7.12	6.44	ND	6.03	6.32	6.03	ND	6.44	ND	ND	ND	ND	ND	ND
S512	ND	7.47	7.11	7.94	ND	7.85	ND	7.47	ND	7.21	ND	7.41	6.90	7.90	ND	7.35	ND	7.03	ND	7.33
S513	ND	6.61	7.11	6.86	ND	6.59	ND	6.47	ND	6.39	ND	6.61	6.90	6.78	ND	6.59	ND	6.25	ND	ND
S516	7.34	6.66	7.80	7.35	6.68	7.19	ND	7.08	ND	6.79	7.21	7.06	6.93	7.36	6.55	6.61	ND	6.33	6.67	6.25
S559	ND	6.67	ND	7.10	6.76	7.28	ND	6.68	ND	6.55	ND	6.36	ND	6.27	ND	6.35	ND	6.21	ND	6.34
S566	ND	ND	ND	ND	ND	6.27	ND	ND	ND	ND	ND	ND	ND	ND	ND	ND	ND	ND	ND	ND
S567	ND	6.67	ND	6.81	ND	7.17	ND	6.69	ND	6.48	ND	6.68	ND	6.70	ND	6.41	ND	6.63	ND	6.71
S570	ND	6.69	ND	6.82	ND	7.14	ND	6.72	ND	6.49	ND	6.70	ND	6.79	ND	6.41	ND	6.64	ND	6.73
S573	ND	6.79	ND	7.02	ND	7.28	ND	6.67	ND	6.63	ND	6.60	ND	6.99	ND	6.26	ND	6.52	ND	6.70
S575	ND	6.79	ND	6.61	ND	6.58	ND	6.58	ND	7.29	ND	6.59	ND	6.63	ND	6.43	ND	6.72	ND	6.52
S598	ND	6.14	ND	6.32	ND	6.69	ND	ND	ND	ND	ND	6.19	ND	ND	ND	ND	ND	6.14	ND	ND
S601	ND	ND	ND	6.61	ND	7.15	ND	ND	ND	6.32	ND	6.04	ND	ND	ND	ND	ND	6.32	ND	6.39
S603	ND	ND	ND	6.51	ND	7.19	ND	ND	ND	6.17	ND	6.19	ND	ND	ND	6.30	ND	6.14	ND	6.39
S605	ND	6.09	ND	6.73	ND	7.73	ND	6.05	ND	6.33	ND	6.39	ND	6.77	ND	6.30	ND	6.18	ND	6.68
T608	ND	ND	ND	6.38	ND	6.75	ND	ND	ND	6.11	ND	ND	ND	6.44	ND	ND	ND	ND	ND	6.50
T609	ND	6.27	ND	6.29	ND	6.45	ND	ND	ND	6.17	ND	ND	ND	6.35	ND	6.13	ND	ND	ND	6.45
S611	6.07	7.11	6.54	7.60	7.37	7.09	6.44	6.89	6.60	7.10	ND	6.45	ND	7.25	ND	6.78	6.28	6.78	6.87	7.47
S612	ND	6.61	ND	7.29	ND	7.59	ND	6.46	ND	6.71	ND	6.59	ND	6.73	ND	6.27	ND	6.65	ND	6.91
S622	7.01	7.88	8.66	8.73	7.77	8.08	7.36	7.77	7.43	7.90	7.30	7.75	8.75	8.88	7.02	7.65	7.01	7.88	6.49	7.83
S625	7.95	8.15	8.95	8.87	8.65	8.41	8.31	8.09	8.23	8.30	8.19	8.11	9.07	9.08	7.98	8.17	8.27	8.27	7.95	8.24
S630	ND	6.37	8.33	7.38	6.37	6.92	ND	ND	ND	6.30	ND	6.49	8.17	7.30	ND	6.19	ND	6.42	ND	ND
S633	ND	6.33	ND	6.57	ND	6.78	ND	6.16	ND	6.23	ND	6.34	ND	6.51	ND	6.32	ND	6.32	ND	ND
S636	ND	6.57	6.45	7.98	6.59	8.13	ND	6.55	ND	6.47	ND	6.70	6.32	7.73	ND	6.65	ND	6.48	ND	ND
S639	ND	ND	6.45	7.72	6.59	7.71	ND	ND	ND	ND	ND	ND	6.32	7.10	ND	ND	ND	ND	ND	6.05
S642	6.32	6.35	7.27	7.78	7.32	7.89	6.50	6.22	6.39	6.31	6.22	6.18	6.87	7.21	6.27	6.40	6.33	6.36	6.63	6.60
S643	ND	6.15	7.69	7.34	8.26	7.36	ND	6.13	ND	6.23	ND	6.07	7.15	7.03	6.20	6.11	ND	6.25	ND	6.05
S652	ND	ND	ND	ND	ND	6.57	ND	ND	ND	ND	ND	ND	ND	ND	ND	ND	ND	ND	ND	ND
S697	ND	6.16	ND	6.48	ND	6.93	ND	ND	ND	ND	ND	ND	ND	ND	ND	ND	ND	ND	ND	ND
S700	ND	8.75	ND	9.17	ND	9.35	ND	8.38	ND	8.74	ND	8.50	ND	8.38	ND	8.38	ND	8.68	ND	8.90

ND (6.00) 11.00
