# Supplementary material for: Comparative phosphorylation map of Dishevelled 3 links phospho-signatures to biological outputs
Source: Cell Commun Signal. 2019 Dec 23;17:170. doi: 10.1186/s12964-019-0470-z (PMC6927192; doi:10.1186/s12964-019-0470-z)
Supplement: Supplementary file 6 [file 12964_2019_470_MOESM7_ESM.docx]

Additional file 6: Table S2

	Analysis	Replicate 1	Replicate 2	Replicate 3	Sample	Experiment
Control 1	Direct	84.21	87.18	75.44	93.66	95.68
	Enriched	75.84	79.62	51.01		
CK1ε	Direct	75.3	70.72	79.76	90.42	
	Enriched	68.83	61.13	69.5		
NEK2	Direct	85.83	76.25	82.32	94.06	
	Enriched	77.46	73.68	61.4		
PLK1	Direct	72.2	79.76	74.9	91.36	
	Enriched	70.58	62.08	57.35		
Aurora A	Direct	78.54	76.79	83.27	93.12	
	Enriched	72.47	68.96	52.09		
Control 2	Direct	82.73	79.62	80.03	92.85	
	Enriched	67.07	69.77	59.92		
TTBK2	Direct	78.68	73.41	77.46	91.36	
	Enriched	76.11	66.4	65.86		
CK2α	Direct	85.16	75.3	82.46	93.25	
	Enriched	74.09	68.96	65.32		
RIPK4	Direct	89.74	75.71	76.79	93.39	
	Enriched	72.33	65.59	52.5		
PKCδ	Direct	73.82	78.41	83.4	91.23	
	Enriched	56.95	68.69	55.74		
